# Oligodendrocyte prosaposin restores subarachnoid haemorrhage-induced consciousness impairment

**DOI:** 10.1038/s12276-026-01764-6

**Published:** 2026-07-02

**Authors:** Haiying Li, Xiang Li, Zhongmou Xu, Chang Cao, Jinxin Lu, Haojie Ding, Lei Bai, Jin Tao, Lu Peng, Jiping Tang, Gang Chen

**Affiliations:** 1 Department of Neurosurgery & Brain and Nerve Research Laboratory, The First Affiliated Hospital of Soochow University, Soochow University, Suzhou, Jiangsu China; 2 Jiangsu Key Laboratory of Drug Discovery and Translational Research for Brain Diseases, Institute of Stroke Research, The First Affiliated Hospital of Soochow University, Suzhou, Jiangsu China; 3 Department of Physiology and Neurobiology, Suzhou Medical College of Soochow University, Suzhou, Jiangsu People’s Republic of China; 4 Physiology and Pharmacology Department of Basic Sciences, Loma Linda University School of Medicine, Loma Linda, CA USA

**Keywords:** Stroke, Stroke

## Abstract

Subarachnoid haemorrhage (SAH) is a life-threatening cerebrovascular event frequently accompanied by consciousness disturbances, yet the underlying mechanisms remain poorly defined. Here, we identify oligodendrocytic prosaposin (PSAP) as a critical regulator of thalamocortical connectivity and consciousness following SAH. Using multimodal approaches, including electroencephalogram and electromyogram recordings, optogenetics, single-nucleus RNA sequencing, patch-clamp recording, and magnetic resonance imaging, we demonstrate that SAH disrupts the central lateral thalamus to medial prefrontal cortex pathway via *Psap* downregulation in oligodendrocytes, leading to impaired PSAP–GPR37 interactions, myelin damage and functional connectivity loss. *Psap* overexpression or recombinant human PSAP administration restored PSAP–GPR37 interactions, preserved myelin structure and rescued central lateral thalamus to medial prefrontal cortex connectivity after SAH, resulting in improved consciousness and spatial memory. Our findings highlight oligodendrocyte dysfunction as a key mechanism underlying SAH-induced disorders of consciousness and identify PSAP as a potential therapeutic target.

## Oligodendrocyte prosaposin restores consciousness after subarachnoid haemorrhage

Subarachnoid haemorrhage (SAH) is a severe cerebrovascular event often leading to consciousness disturbances due to disrupted thalamocortical pathways. This study explores the impact of SAH on the central lateral thalamus to medial prefrontal cortex neural pathway and the potential therapeutic role of prosaposin (PSAP). Using electroencephalogram recordings, optogenetics and single-nucleus RNA sequencing, researchers found that SAH disrupts the central lateral thalamus–medial prefrontal cortex pathway, leading to impaired consciousness in mice. The study identified a significant reduction in PSAP, a protein crucial for myelin integrity, in oligodendrocytes following SAH. Overexpression of PSAP or administration of recombinant human PSAP restored neural connectivity and improved consciousness and cognitive function. These findings suggest that PSAP–GPR37 signalling is vital for maintaining neural pathway integrity and highlight PSAP as a promising therapeutic target for SAH-induced consciousness disorders.

This summary was initially drafted using artificial intelligence, then revised and fact-checked by the author.

## Introduction

Subarachnoid haemorrhage (SAH) is a critical cerebrovascular event with high mortality and long-term neurological impairments, including consciousness disturbances^1^. Several pathophysiological mechanisms contribute to consciousness disturbances following SAH, including increased intracranial pressure, cerebral vasospasm, neuroinflammation and transient intracranial circulatory arrest^2^. Loss of consciousness at symptom onset is an important manifestation of early brain injury after SAH and a predictor of death or poor functional outcome at 12 months^1^.

Functional magnetic resonance imaging (MRI) and electrophysiological studies have shown that consciousness relies on large-scale interactions between the thalamus and cortex^3^. Research shows that, in patients with impaired consciousness, disruption in thalamic cortical functional connections is closely related to a decline in consciousness levels^4^. Among the neural pathways critical for maintaining consciousness, the central lateral thalamus (CL) to medial prefrontal cortex (mPFC) neural pathway has been identified as a key player^5^. Stimulation of the CL during anaesthesia has been found to restore wakefulness, indicating that thalamocortical pathways are essential for maintaining conscious states^3^. Damage to the mPFC has been shown to result in profound impairments in consciousness^6^. Studies have also demonstrated that neural activity within thalamocortical networks is vital for sensory processing, memory, decision-making and action, with thalamic neurons receiving convergent inputs from multiple cortical regions^7^. These findings underscore the importance of the CL→mPFC neural pathway in consciousness maintenance and provide a foundation for investigating how SAH may disrupt this neural pathway.

White matter, composed of myelinated axons that facilitate communication between brain regions, is critical for normal neurological function. Damage to white matter can lead to neural pathway dysfunction and consciousness disorders^8^. Research shows that, after stroke, disruption of neural pathway function is primarily due to structural damage in white matter, rather than localized cortical injury, which impairs brain connectivity and leads to consciousness deficits^9^. In patients with SAH, an increase in white matter diffusion rate within 2 weeks of onset correlates with cognitive impairment 3 months later^10^. Further investigation into the regulatory mechanisms of white matter injury is essential for developing therapeutic strategies to improve SAH prognosis.

Oligodendrocytes, a critical component of white matter, play an essential role in maintaining the stability and function of neural pathways by forming myelin sheaths and providing metabolic support^11^. Abnormalities in oligodendrocyte function, including myelin degeneration, have been associated with impaired consciousness recovery in various neurological disorders^12^. However, the role of oligodendrocyte dysfunction and its impact on consciousness disorders in SAH remains largely unexplored. Prosaposin (PSAP) protects myelin integrity and prevents oligodendrocyte death under pathological conditions, and its reduction has been linked to white matter damage^13–15^. However, the role of PSAP in SAH-induced white matter damage and consciousness disorders remains unexplored.

This study used electroencephalogram (EEG) and electromyogram (EMG) recordings to demonstrate that SAH induced consciousness impairment in mice. Additionally, patch-clamp and optogenetic techniques revealed that SAH disrupted the CL→mPFC neural pathway. Single-nucleus RNA sequencing (snRNA-seq) further identified oligodendrocyte heterogeneity and revealed a reduction in PSAP — a multifunctional protein essential for neuronal and glial function — following SAH. This study aimed to investigate the effects of SAH on the CL→mPFC neural pathway and assess the potential of PSAP as a therapeutic target for consciousness disorders following SAH.

## Materials and methods

### Animals

Adult male C57BL/6J mice (8–10 weeks old, 22–26 g) were obtained from Hangzhou Enlighten the Truth Laboratory Animal Technology Co., Ltd. (Hangzhou, China) and housed under standard laboratory conditions (12-h light/dark cycle, ambient temperature 22 ± 2 °C, with ad libitum access to food and water). All animal procedures were approved by the Institutional Animal Care and Use Committee of the First Affiliated Hospital of Soochow University and conducted in accordance with the National Institutes of Health Guide for the Care and Use of Laboratory Animals.

### SAH induction

SAH was induced using the endovascular perforation model as previously described^16^. Briefly, mice were anaesthetized with isoflurane, and a nylon filament was inserted through the external carotid artery to perforate the bifurcation of the anterior and middle cerebral arteries. Sham-operated mice underwent the same procedure without vessel perforation.

### Human sample collection and processing

This study recruited 12 patients with aneurysmal acute SAH undergoing surgery and 6 patients undergoing brain tumour surgery as controls between January 2019 and January 2025. The study was approved by the Ethics Committee of the First Affiliated Hospital of Soochow University (Approval No. 2024-609) and registered in the Medical Research Registration and Information System (Registration No.: MR-32-25-034933).

All plasma and brain tissue samples were collected under standardized surgical protocols, with written informed consent obtained prior to collection, in accordance with the 2024 revision of the World Medical Association Declaration of Helsinki. All samples were de-identified before analysis to ensure donor confidentiality.

Brain tissue specimens were obtained intraoperatively from two distinct pathological contexts: (1) injured white matter adjacent to haemorrhage sites in patients with SAH, and (2) peritumoural regions in patients with a brain tumour. Aneurysmal SAH was diagnosed by computed tomography and digital subtraction angiography performed within 24 h of admission.

Exclusion criteria were: (1) other intracranial diseases; (2) a history of autoimmune disorders, acute or chronic infections, haematological diseases, or major surgery within the past year; and (3) use of corticosteroids or immunosuppressants within the past 6 months.

Detailed clinical characteristics, including age, sex, aetiology, bleeding volume, Hunt and Hess grade, and sample type, are listed in Supplementary Table 1.

### EEG and EMG recordings

Mice were anaesthetized and mounted in a stereotaxic apparatus and then implanted for EEG and EMG recordings. Briefly, screw electrodes were inserted into the skull of mice to measure cortical EEG, and stainless-steel electrodes were implanted in dorsal neck muscle to measure EMG. After 1 week of EEG and EMG implantation, the mice were acclimated to the custom-made device for 3 days. For the SAH group, after electrode implantation and acclimatization to the equipment in the same manner, SAH was induced, and recordings were conducted 48-h post-SAH. The recording process started at 7:00 a.m. (ZT0) to 7:00 a.m. (ZT24) at day 13. Cortical EEG and EMG signals were amplified, filtered (high-pass >0.5 Hz; band-pass 5–45 Hz), and digitized at 1000 Hz using a tethered data acquisition system (Medusa, Bio-Signal Technologies, China). The power of the d (0.5–4 Hz), q (5–8 Hz), and a (9–14 Hz) bands as well as the d band ratio were calculated, and the EMG signal was scored. The sleep stages in the recordings were scored by artificial intelligence-driven software, Lunion Stage, a type of deep learning model for extracting spectrogram features from the EEG channel and movement features from the EMG channel. These data were analysed in 4-s epochs to define the vigilance states of wake, non-rapid eye movement (NREM) and rapid eye movement (REM) based on their spectral and waveform properties. The scored results were examined and manual adjustments were made as needed, followed by statistical analysis performed on different vigilance states and groups.

Inclusion and exclusion criteria: animals were assigned to experimental groups and included in the final statistical analysis based on predefined inclusion and exclusion criteria. Prior to the formal experiment, sleep–wake cycle monitoring was conducted. Mice exhibiting wakefulness for more than 60% or less than 40% of the monitoring period were excluded to ensure consistent baseline sleep–wake patterns across groups. Following the establishment of the SAH model, only mice exhibiting wakefulness below 40% were included for further analysis, representing approximately 58% of all SAH mice.

### Fibre photometry recording

For fibre-optic implantation, a 2.5 mm optic fibre (Thinkerbiotech) was positioned 0.2 mm above the centre of the injection site and secured to the skull with dental acrylic. Following viral injection and optical fibre cannula implantation, mice were allowed to recover for 2 weeks. After recovery, the cannulas were connected to the fibre recording system (QAXK-LASER-Y1, Thinkerbiotech), and mice were habituated for 1 week. Recordings were conducted 3 weeks post-viral transfection. ChrimsonR opsin expressed in the upstream CL nuclei was activated using 589 nm yellow light (10 ms pulses, 20 Hz, 5 mW), with each light stimulation lasting 5 s, followed by a 25-s interval. Each mouse underwent three stimulation cycles, while calcium signals from GCaMP6s in the downstream mPFC nuclei were recorded simultaneously (470 nm, 20 mW). If mice exhibited signs of agitation or heightened alertness during the recording, they were first allowed to acclimate to the environment for 1 h, and baseline changes were recorded. Photometry data were exported as MATLAB files for further analysis. Each mouse underwent three experimental sessions, and fluorescence changes (ΔF/F) were calculated as (F-F0)/F0. The ΔF/F values were displayed as heatmaps or averaged traces, with F0 representing the mean baseline fluorescence signal from a 1-s window prior to the onset of light stimulation. The normalized area under the curve (AUC) was calculated by summing the fluorescence changes during the light stimulation period and dividing by the duration of the behavioural phase. ΔF/F was represented as heatmaps or averaged traces, with shaded areas indicating the standard error of the mean.

### Electrophysiological recordings

Mice were deeply anaesthetized with isoflurane and perfused with ice-cold oxygenated cutting solution. The entire brain was quickly dissected and placed in ice-cold oxygenated cutting solution. Coronal brain slices (300 µm thick) containing the target region were prepared using a vibratome (VTA-1200S; Leica) and incubated in cutting solution at 33 °C for 15 min before being transferred to the incubation solution. Slices were incubated at room temperature (24–26 °C) for at least 1 h before being transferred to a fluorescence microscope (BX51Wl; Olympus) for recordings. All neuronal recordings were performed in artificial cerebrospinal fluid, with the bath solution continuously oxygenated (95% O_2_, 5% CO_2_) and maintained at ~33 °C using a temperature control system to ensure cell viability. After identifying the target region under a 10× objective lens, the microscope lens was switched to 40× for whole-cell patch-clamp recordings. Borosilicate glass electrodes (BF150-86-10; Sutter Instrument) were pulled using a P97 puller (Sutter Instrument), resulting in a resistance of 3–5 MΩ, and then filled with electrode solution for neuronal recordings. Electrophysiological recordings were performed using a Multiclamp 700B amplifier (Molecular Devices) and a DigiData 1440A analogue-to-digital converter (Molecular Devices). Signals were filtered at 10 kHz and digitized at 10 kHz (MICRO3 1401, Cambridge Electronic Design). Data acquisition and analysis were conducted using Clampfit 10.6 software (Cambridge Electronic Design). Strict quality control criteria were applied to the recorded cells: series resistance <25 MΩ, input resistance >300 MΩ and leak current <50 pA. Cells not meeting these criteria were excluded from further analysis.

### Viral injection

Mice were deeply anaesthetized and head-fixed in a stereotaxic frame (RWD Instruments). Viral vectors were injected into one of the following regions using a 10-μl Hamilton microsyringe: the CL thalamus (anteroposterior (AP): –1.72 mm from bregma, mediolateral (ML): ±0.46 mm, dorsoventral (DV): –2.55 mm from the brain surface), the mPFC (AP: –1.94 mm, ML: 0.85 mm, DV: –1.20 mm) or the corpus callosum (AP: –1.06 mm, ML: ±0.70 mm, DV: –1.40 mm). After injection, the needle was left in place for ~10 min to prevent reflux.

HSV-tdTomato (50 nl; H01002, BrainVTA) was injected 48-h post-SAH, and brain tissue was collected 3 days later. AAV-hSyn-EGFP(2/Re) (50 n; PT-1990, BrainVTA) was injected 48-h post-SAH, and tissue was collected 21 days later. AAV-CaMKIIα-ChrimsonR (50 nl; PT-4285, BrainVTA), AAV-hSyn-GCaMP6s (50 nl; PT-0145, BrainVTA), AAV-MBP-PSAP-mCherry (200 nl; PT-1515, BrainVTA), and rAAV-MBP-EGFP-shRNA(PSAP)-WPREs (200 nl; PT-16167, BrainVTA) were injected and allowed to express for 21 days.

### Oligodendrocyte-specific *Gpr37* knockdown

PLP-CreERT2 transgenic mice were purchased from Cyagen Biosciences (Suzhou, China) and used to achieve oligodendrocyte-specific gene manipulation. To induce Cre recombinase activity, tamoxifen (Sigma-Aldrich, T5648) was dissolved in corn oil at a concentration of 20 mg/ml and administered intraperitoneally at 75 mg/kg body weight once daily for 5 consecutive days, starting 2 weeks before SAH surgery.

For oligodendrocyte-specific *Gpr37* knockdown, Cre-dependent adeno-associated viral (AAV) vectors (rAAV-CMV-DIO-(mCherry-U6)-shRNA(*Gpr37*)) or control vectors (rAAV-CMV-DIO-(mCherry-U6)-shRNA(scramble) (viral titre: 1 × 10¹³ vg/ml, BrainVTA) were bilaterally injected into the corpus callosum (AP: –1.06 mm, ML: ±0.70 mm, DV: –1.40 mm from bregma) at a volume of 200 nl per site. The injection was performed 3 weeks before SAH surgery to allow sufficient time for viral expression and *Gpr37* knockdown.

### Magnetic resonance imaging

Mice were anaesthetized using a gas mixture (induction: 3% isoflurane with 1 l/min O_2_; maintenance: 1.5% isoflurane with 1 l/min O_2_) and MRI scanning was performed using a 9.4 Tesla animal scanner (Bruker Biospin). T2-weighted images were acquired using the RARE sequence. White matter signal values were obtained from the raw DICOM images using 3D Slicer software.

### Immunofluorescence staining

Mouse brain samples were collected following transcardial perfusion with phosphate-buffered saline and 4% paraformaldehyde (PFA). Brains were post-fixed in PFA, cryoprotected in graded sucrose solutions, embedded, and sectioned at 20 μm thickness using a cryostat. Free-floating sections were subjected to antigen retrieval, permeabilized and blocked in a serum-containing buffer, followed by overnight incubation with primary antibodies at 4 °C. The sections were then incubated with fluorescent secondary antibodies at room temperature, counterstained with 4′,6-diamidino-2-phenylindole (DAPI) and mounted. Immunofluorescence images were acquired under identical settings across all experimental groups.

Human brain samples were obtained as paraffin-embedded sections. Following deparaffinization and rehydration through xylene and ethanol gradients, antigen retrieval was performed using heat-induced sodium citrate buffer. Sections were then permeabilized, blocked and incubated with primary antibodies overnight at 4 °C, followed by incubation with fluorescent secondary antibodies. Nuclei were stained with DAPI, and images were acquired under consistent conditions across all samples. Primary and secondary antibodies used in this study are listed in Supplementary Table 2.

### RNAscope FISh

RNAscope fluorescent in situ hybridization (FISH) was performed using the RNAscope Multiplex Fluorescent v2 Assay (Advanced Cell Diagnostics, ACD) according to the manufacturer’s instructions with minor modifications. Briefly, fresh-frozen coronal brain sections (20 μm) were mounted on SuperFrost Plus slides, fixed in 4% PFA for 15 min at 4 °C, and dehydrated through a graded ethanol series. Sections were pretreated with hydrogen peroxide for 10 min and Protease IV for 30 min at room temperature. A target probe against mouse *Psap* mRNA (ACD, Cat# 545241) was hybridized for 2 h at 40 °C in a HybEZ oven, followed by sequential signal amplification steps (Amp1–Amp3). The *Psap* probe signal was visualized using Opal 570 fluorophore (red). Following RNAscope detection, sections were subjected to immunofluorescence staining for the mature oligodendrocyte marker CC1 (Abcam, ab16794, 1:200) and the neuronal marker NeuN (Abcam, ab104224, 1:500), with appropriate fluorescent secondary antibodies. Images were acquired using a confocal microscope under identical settings across all experimental groups.

### Single-nucleus processing and library preparation

The 10x Genomics Chromium is based on GemCode technology and allows high-throughput single-cell 3’ mRNA quantitative analysis that describes intercellular heterogeneity.

The nuclei suspension was stained with 0.4% trypan blue to assess cell viability under microscopic observation. Cells with greater than 80% viability were qualified for the library construction process. The prepared single-cell suspension was then partitioned into Gel Beads in Emulsions in the automated Chromium Controller, and mRNAs were then reverse transcribed into complementary DNAs (cDNAs). The Gel Beads in Emulsions were broken and purified for a fixed period. A certain amount of cDNA amplified products was taken and subjected to fragmentation, end repair and addition of an ‘A’ base at the 3’-end of each strand. Subsequently, the products were purified. cDNAs were subjected to adaptor ligation and purified. The cDNA with an adaptor was amplified via PCR, and the products were subjected to library quality control. Single-stranded PCR products were produced via denaturation. The reaction system and programme for circularization were subsequently configured and set up. Single-stranded cyclized products were produced, while uncyclized linear DNA molecules were digested. Single-stranded circular DNA molecules were replicated via rolling-circle amplification, and a DNA nanoball containing multiple copies of DNA was generated. Sufficient quality DNA nanoballs were then loaded into patterned nanoarrays using a high-intensity DNA nanochip technique and sequenced through combinatorial probe–anchor synthesis.

### Sequencing, pre-processing and quality control

All samples were processed in a single flow cell. Reads were demultiplexed and aligned to the *Mus musculus* reference genome refdata-gex-mm10-2020-A via Cellranger v.6.1.2, incorporating intronic reads to capture unspliced transcripts, following the developer’s instructions. Subsequent processing of the Cellranger output was performed using R and R Studio (R version 4.1.2, The R Foundation, Vienna, Austria), with specific packages used as needed. All snRNA-seq analyses were carried out within Seurat (v.5.0.1), unless otherwise specified, following the developer’s guidelines. Quality control was performed to exclude low-quality or stressed cells. Cells were retained for downstream analysis if they had between 300 and 7000 detected genes, less than 10% mitochondrial gene content, less than 3% haemoglobin gene expression, and fewer than 100,000 total Unique Molecular Identifier counts. Unique Molecular Identifier count matrices for each dataset were generated and converted into Seurat objects. snRNA-seq data were normalized using the ‘NormalizeData’ function and scaled with ‘ScaleData’ to centre and standardize gene expression. The processed data were subsequently used for integration, dimensionality reduction and clustering analyses. Doublets were detected and excluded using DoubletFinder v2.0.3, based on the developer’s recommendations^17^.

### Dataset integration

Integration across samples was performed using canonical correlation analysis implemented in the ‘FindIntegrationAnchors’ function, with 2000 shared variable features used to identify anchors. Batch correction was then achieved using the ‘IntegrateData’ function to generate an integrated expression matrix suitable for downstream analyses.

### Clustering and subclustering of cell types

Principal component analysis was carried out using the Seurat function ‘RunPCA’. The top 25 principal components, selected based on variance explained, were used to construct a shared nearest neighbour graph with ‘FindNeighbors’, followed by Louvain clustering using ‘FindClusters’ at a resolution of 1.2. Dimensionality reduction for visualization was performed using uniform manifold approximation and projection (UMAP) with the ‘RunUMAP’ function. For subclustering, selected clusters were isolated, split by sample, and normalization and integration were repeated using the same parameters. Subsequently, principal component analysis, UMAP and Louvain clustering were reapplied to the pre-processed and reintegrated subsets to derive sub-clusters.

### Differential gene expression analysis

The MAST statistical framework^18^ within Seurat’s ‘FindAllMarkers’ and ‘FindMarkers’ functions was used for differentially expressed gene calculations to identify cluster markers and between-group differences in gene expression. Genes expressed in at least 1% of cells and with a minimum log-fold change of 0.01 were considered. Differentially expressed genes were further filtered by statistical significance (*P* <0.05) and an absolute average log2 fold change greater than 0.25.

### SCENIC analysis

The identification of potential transcription factors was conducted through gene co-expression analysis, implemented using the GENIE3 (GEne Network Inference with Ensemble of trees) algorithm. GENIE3 takes a single-cell gene expression matrix as the input file, with transcription factors as the input variables, and each target gene as the output. It constructs P random forests (P representing the number of genes in the matrix) and computes an importance score for each transcription factor in relation to each gene, thereby generating transcription factor–gene co-expression modules. Relationships between genes with importance scores below a set threshold were eliminated, and modules containing fewer than 50 genes were filtered out. Enrichment analysis of transcription factor-binding motifs and identification of target genes (regulons) was performed using the RcisTarget package. RcisTarget identifies transcription start sites for genes in the provided gene list and detects frequently occurring DNA motifs near these transcription start sites. It then searches a cross-species genomic database of motif information to identify motifs associated with the target transcription factor that have a normalized enrichment score >3.0. SCENIC uses AUCell to calculate the activity of regulons. AUCell computes the AUC to quantify regulon activity based on gene expression levels.

### Cell trajectory-based pseudotime inference analysis

Pseudotime trajectory analysis was conducted using the R package monocle (version 2.22), with default settings unless otherwise specified. Genes used for pseudotime ordering were selected via the DifferentialGeneTest function. Dimensionality reduction and cell ordering along the pseudotime trajectory were performed using the DDRTree method. Branch analysis was conducted with the BEAM function. Genes showing significant changes at branch points (*P* <0.01) were classified into five clusters based on distinct expression patterns across two different cell states. Heatmaps were generated based on pseudotime-dependent differentially expressed genes, and Gene Ontology enrichment analysis was performed on the top 100 genes in each cluster, ranked by q-value, which reflects the gene’s association with pseudotime.

### Cell–cell communication analysis

To investigate cell–cell communication within the microenvironment, CellChat was used to predict ligand–receptor (molecules that bind and transmit signals) interactions between oligodendrocytes and other cell populations^19^. In summary, normalized expression data from each sample were input into CellChat, and pre-processing was performed using the functions ‘identifyOverExpressedGenes’, ‘identifyOverExpressedInteractions’ and ‘projectData’. The core functions, including ‘computeCommunProb’ (to estimate the probability of communication between cells), ‘computeCommunProbPathway’ (to predict pathway-level communication) and ‘aggregateNet’ (to summarize the overall communication network), were used to infer these cell–cell networks. Significant interactions were considered when *P* values were ≤0.05. Finally, the ‘netAnalysis_signalingRole_heatmap’ function identified the sender (signal-emitting) and receiver (signal-receiving) cells in the network.

### Proximity ligation assay

Free-floating sections underwent antigen retrieval, permeabilization and blocking according to the Duolink PLA protocol (MilliporeSigma, DUO94102). After overnight incubation with primary antibodies against PSAP and GPR37 at 4 °C, sections were incubated with PLA probes, followed by ligation and rolling-circle amplification. Nuclei were counterstained with DAPI, and signals were visualized under a fluorescence microscope. All samples were processed in parallel using identical conditions, and quantification was performed using ImageJ.

### ELISA

Plasma levels of PSAP were quantified using mouse (Finetest, EM2515) and human (Finetest, EH3669) ELISA kits according to the manufacturers’ instructions. All assays were performed in technical duplicates, and absorbance was measured at 450 nm using a microplate reader.

### Electron microscopy imaging

Mice were anaesthetized and perfused with glutaraldehyde. Small corpus callosum tissue blocks (~1 mm³) were dissected and fixed in 2.5% glutaraldehyde at 4 °C, followed by post-fixation in 1% osmium tetroxide. Samples were dehydrated in a graded ethanol series, transitioned through propylene oxide, and embedded in epoxy resin. Resin blocks were polymerized at 60 °C and sectioned using an ultramicrotome. Ultra-thin sections (~70 nm) were collected on copper grids, stained with uranyl acetate and lead citrate, and imaged using a transmission electron microscope.

### Stereotaxic injection of recombinant protein

Recombinant human PSAP (HuPSAP) protein (Abcam, ab167924) was reconstituted in deionized water to a final concentration of 0.5 mg/ml, aliquoted and stored at –80 °C until use. Mice were anaesthetized with isoflurane and positioned in a stereotaxic frame. A small burr hole was drilled over the corpus callosum (AP: –1.06 mm, ML: ± 0.70 mm, DV: –1.40 mm from bregma), and 1 µl of recombinant protein solution was injected bilaterally using a microsyringe.

### Morris water maze

Spatial learning and memory were assessed using the Morris water maze test as previously described, with minor modifications^20,21^. A circular pool (90 cm diameter) filled with water maintained at 21–22 °C was used. After 1 day of visible platform training, mice underwent 5 consecutive days of hidden-platform training (12 trials/day), with the platform submerged 1.0 cm below the surface. Each trial ended upon reaching the platform or after 120 s. On day 6, a probe trial was conducted by removing the platform and allowing mice to swim freely for 120 s. Escape latency, swim distance, and time spent in each quadrant were recorded and analysed.

### Statistical analysis

All statistical analyses were conducted using GraphPad Prism (version 9.0.0). Sample size was determined using G*Power software (version 3.1.9.2). Data are presented as mean ± standard deviation. Statistical significance was defined as *P* <0.05. Detailed statistical results and methods are provided in Data File S1.

## Results

### SAH disrupts consciousness and impairs the CL→mPFC neural pathway in mice

**Fig. 1:**
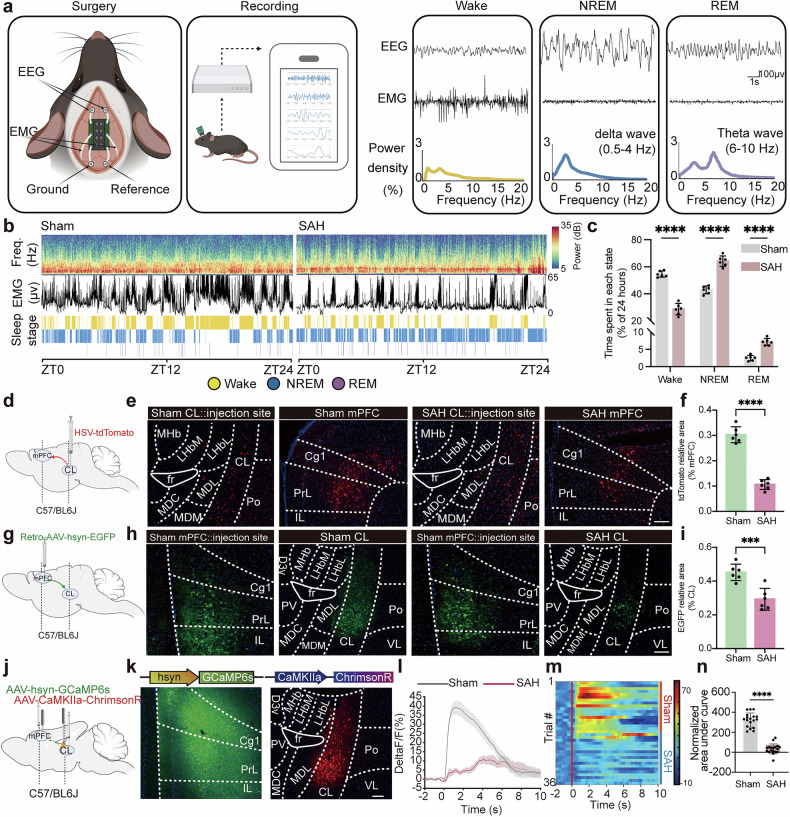
SAH disrupts consciousness and impairs the CL→mPFC neural pathway in mice. Schematic of electroencephalogram and electromyogram (EEG/EMG) electrode implantation and recording setup (part **a**). Representative EEG/EMG traces and power spectra for wake, non-rapid eye movement (NREM) and rapid eye movement (REM) sleep states. Representative EEG spectrograms, EEG/EMG traces, and sleep state distributions over a 24-h cycle in sham and subarachnoid haemorrhage (SAH) mice (part **b**). Quantification of time spent in wake, NREM and REM states (*n* = 6, ****P <0.0001) (part **c**). Schematic of HSV-tdTomato viral injection into the central lateral thalamus (CL) for anterograde tracing (part **d**). Representative fluorescence images showing tdTomato-labelled projections from the CL to the medial prefrontal cortex (mPFC) in sham and SAH mice (part **e**; scale bar: 200 μm). Quantification of tdTomato-positive area in the mPFC, expressed as a percentage of total mPFC area (*n* = 6, ****P<0.0001) (part **f**). Schematic of retrograde viral tracing with AAV2-retro-hsyn-EGFP injected into the mPFC (part **g**). Representative fluorescence images showing EGFP-labelled CL neurons in sham and SAH mice (part **h**; scale bar: 200 μm). Quantification of EGFP-positive area in the CL, expressed as a percentage of total CL area (*n* = 6, ***P<0.001) (part **i**). Schematic of optogenetic manipulation using AAV-hsyn-GCaMP6s (mPFC) and AAV-CaMKIIα-ChrimsonR (CL) with fibre photometry recording (part **j**). Representative fluorescence images of GCaMP6s and ChrimsonR expression in sham and SAH mice (part **k**; scale bar: 200 μm). GCaMP6s fluorescence traces and corresponding heatmap showing optogenetic stimulation-induced calcium responses in sham and SAH groups (parts **l** and **m**). Quantification of normalized area under the curve for optogenetic responses in sham vs SAH mice (*n* = 6, ****P<0.0001) (part **n**).

To establish the SAH model, we performed endovascular perforation in adult C57BL/6J male mice, with sham-operated controls receiving identical surgical procedures but excluding vessel rupture (see Methods). To evaluate the functional consequences of SAH on consciousness, EEG/EMG-based sleep–wake analysis was performed on mice with implanted cortical and cervical electrodes (Fig. 1a). The wake period was defined as the phase characterized by desynchronized, low-amplitude EEG rhythms and episodic bursts of increased EMG activity. NREM sleep was identified by synchronized, high-amplitude, low-frequency (0.5–4 Hz, δ) EEG activity and lower EMG activity compared to the wake period, without phasic bursts. REM sleep was defined as the period featuring prominent θ (6–10 Hz) rhythms and minimal EMG activity^22–24^. Compared with sham mice, SAH mice displayed significantly reduced wakefulness throughout the light–dark cycle (Fig. 1b, c), with a corresponding increase in NREM and REM sleep.

Consciousness relies on large-scale thalamocortical and corticocortical connectivity^3,25,26^. Previous studies have identified the CL as a key structure for maintaining consciousness^3^. To evaluate whether SAH disrupts CL-mediated cortical connectivity, multisynaptic neural pathway tracing was performed using an anterograde herpes simplex virus (HSV). The virus was injected into the CL region 48 h post-modelling (Fig. 1d), and brain sections were collected 3 days later. In sham mice, bright red fluorescent tdTomato signals were detected in the mPFC neurons, while in SAH mice, the fluorescent signal in the mPFC was significantly reduced, indicating impaired CL→mPFC connectivity (Fig. 1e, f). To further validate these findings, a retro-AAV-hSyn-EGFP was injected into the mPFC of sham and SAH mice 48 h post-modelling (Fig. 1g). At 21 days post-injection, strong EGFP fluorescence was detected in the CL of sham mice, while fluorescence intensity was markedly diminished in SAH mice (Fig. 1h, i), confirming disrupted reciprocal connectivity between the CL and mPFC. Additionally, we used optogenetics to examine functional impairments in the CL→mPFC neural pathway. Given that CL neurons are primarily glutamatergic^27,28^, we targeted these neurons using an AAV carrying the CaMKIIα-ChrimsonR construct for optogenetic activation, along with an AAV-Syn-GCaMP6s construct to measure calcium activity in the mPFC (Fig. 1j). Optogenetic stimulation (589 nm, 20 Hz, 10 ms pulses, 5 mW, 5 s on and 25 s off cycle) was applied to activate ChrimsonR-expressing CL neurons while recording calcium transients in the mPFC. In sham mice, stimulation of the CL induced a robust increase in calcium signal (quantified by AUC) in the mPFC. However, in SAH mice, optogenetic activation failed to elicit significant calcium responses, suggesting profound disruption of functional connectivity within the CL→mPFC neural pathway (Fig. 1k–n).

### SAH disrupts CL→mPFC neural pathway connectivity

**Fig. 2:**
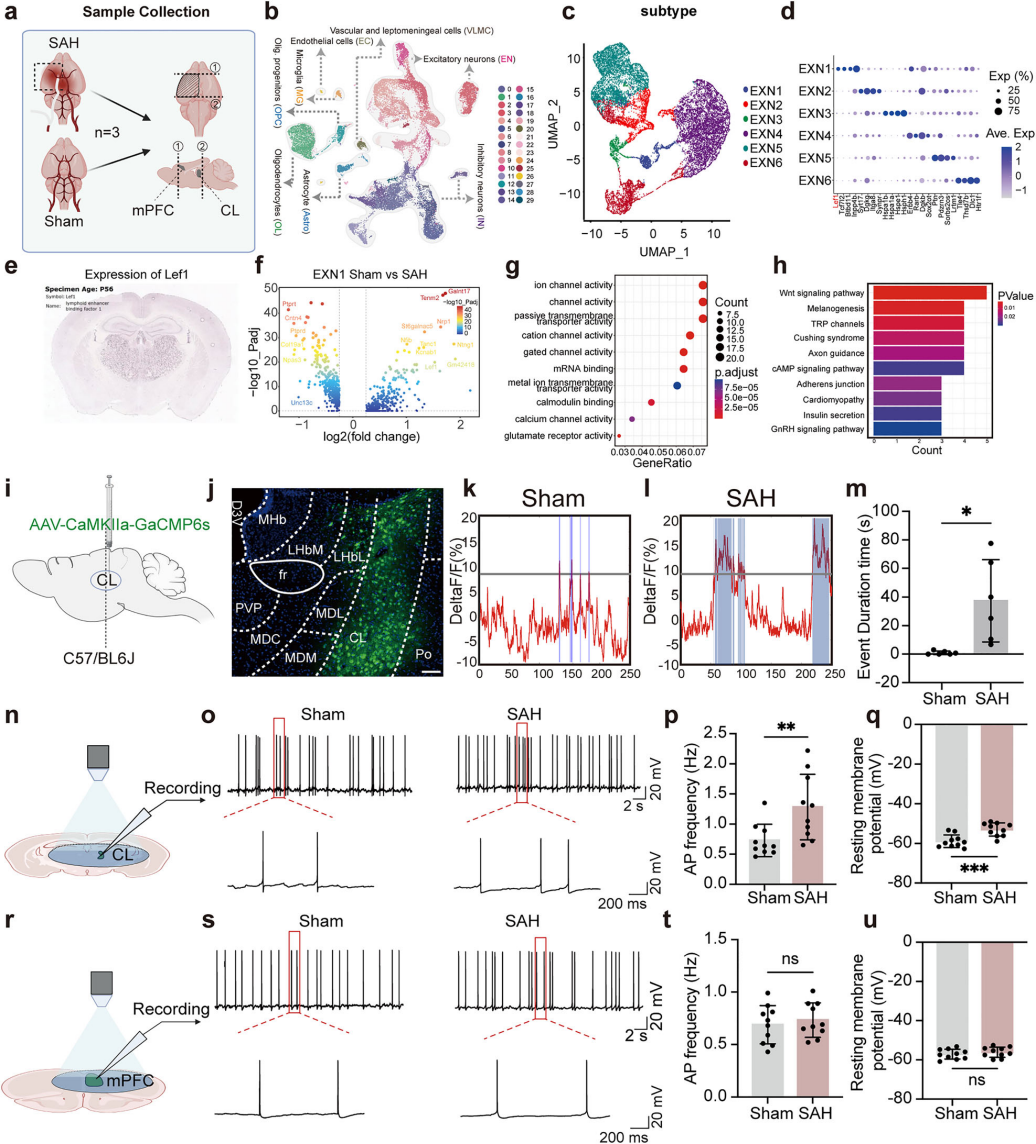
snRNA-seq and electrophysiology reveal SAH-induced transcriptional remodelling and hyperexcitability in thalamic excitatory neurons. Schematic overview of sample collection for single-nucleus RNA sequencing (snRNA-seq) from the haemorrhagic hemisphere of subarachnoid haemorrhage (SAH) and sham mice (part **a**). Uniform manifold approximation and projection (UMAP) visualization of cell-type clusters identified by snRNA-seq, including excitatory neurons (EXN), inhibitory neurons, oligodendrocytes, oligodendrocyte precursor cells, astrocytes, vascular and leptomeningeal cells, and endothelial cells (part **b**). UMAP feature plots of excitatory neuron clusters (Clusters 1–6), demonstrating transcriptomic heterogeneity (part **c**). Dot plot showing the percentage of cells expressing key marker genes in each EXN subtype (EXN1–EXN6), with expression intensity indicated by colour scale (part **d**). Allen Brain Atlas image showing *Lef1* expression predominantly in the thalamus (part **e**). Volcano plot of differentially expressed genes in EXN1 (part **f**). Gene Ontology functional enrichment analysis of upregulated differentially expressed genes in EXN1 (part **g**). Reactome pathway enrichment analysis of upregulated differentially expressed genes in EXN1 (part **h**). Schematic of the experimental setup for fibre photometry recording, with AAV-CaMKIIα-GCaMP6s injected into the central lateral thalamus (CL) (part **i**). Representative fluorescence image of GCaMP6s expression in the CL (part **j**; scale bar: 200 μm). Example calcium fluorescence traces from sham and SAH mice (parts **k** and **l**). Quantification of calcium transient duration (*n* = 6, *P<0.05) (part **m**). Schematic representation of patch-clamp recording sites in the CL and medial prefrontal cortex (mPFC), respectively (parts **n** and **r**). Representative action potential traces from sham and SAH neurons in the CL and mPFC (parts **o** and **s**). Quantification of action potential (AP) frequency in the CL and mPFC (*n* = 10, **P<0.01) (parts **p** and **t**). Resting membrane potential measurements (*n* = 10, ***P<0.001) (parts **q** and **u**). ns, not significant.

To investigate the molecular mechanisms underlying SAH-induced CL→mPFC neural pathway damage, snRNA-seq was performed on mouse brains dissected from the haemorrhagic hemisphere, spanning from the mPFC origin (bregma 2.34 mm) to the CL termination (bregma –2.18 mm), 12 h after SAH modelling (Fig. 2a and Supplementary Fig. 1a; *n* = 3). A total of 26,589 nuclei were profiled, with 13,734 from sham brains and 12,855 from SAH brains. Cell types, including excitatory neurons (EXN), inhibitory neurons, oligodendrocytes, astrocytes, vascular and leptomeningeal cells, and endothelial cells, were identified and visualized using UMAP (Fig. 2b). The accuracy of cell-type classification was validated by a heatmap of differentially expressed genes across annotated cell types (Supplementary Fig. 1b) and a bubble plot showing marker-based cell classification (Supplementary Fig. 1c).

Further snRNA-seq analysis revealed transcriptional heterogeneity among excitatory neurons, which were clustered into six distinct groups (Clusters 1–6) based on their unique transcriptomic signatures (Supplementary Fig. 2a). These clusters were further categorized into six excitatory subtypes, EXN1–EXN6 (Fig. 2c, d). SCENIC analysis identified *Lef1* as a key transcriptional regulator within the EXN1 subpopulation (Supplementary Fig. 3a, b). Notably, EXN1 was enriched for *Lef1*, a gene predominantly expressed in the thalamus according to the Allen Brain Atlas^29^ (Fig. 2e and Supplementary Fig. 3c). In our dataset, *Lef1* expression was highly specific to EXN1 (Supplementary Fig. 2e–g), and this subtype-specific pattern was further supported by elevated LEF1 protein levels in the thalamus of SAH mice compared to sham controls (Supplementary Fig. 3d–f), while NeuN-positive neuronal density in the CL remained unchanged (Supplementary Fig. 3g). Collectively, these multimodal findings define EXN1 as a transcriptionally distinct excitatory neuronal subtype within the thalamus.

To investigate the impact of SAH on the EXN1 subtype, we performed KEGG and Reactome pathway enrichment analyses on genes upregulated in EXN1 neurons in SAH mice compared to sham controls. These analyses revealed that SAH-induced upregulated genes in EXN1 were primarily associated with ion channel activity and the Wnt signalling pathway, rather than wth pathways related to neuronal injury or cell death (Fig. 2f–h and Supplementary Fig. 2b–d). Given the involvement of ion channels and Wnt signalling in neuronal excitability, we further examined whether SAH affects calcium dynamics within the CL. A calcium-sensitive fluorescent probe, GCaMP6s, was injected into the CL, followed by the implantation of optical fibres for fibre photometry recording (Fig. 2i, j). Compared to sham mice, SAH mice exhibited significantly elevated spontaneous calcium signals in the CL (Fig. 2k–m), indicating increased neuronal activity. To further assess neuronal excitability, we performed patch-clamp electrophysiology in both the CL (origin site) and mPFC (termination site) ends of the CL→mPFC pathway (Supplementary Fig. 4a and Fig. 2n, r). In the CL, SAH neurons exhibited significantly higher action potential frequency and a more depolarized resting membrane potential compared with sham controls (Fig. 2o–q). In contrast, mPFC neurons showed no significant differences in either action potential frequency or resting membrane potential between SAH and sham groups (Fig. 2s–u). Electrophysiological recordings revealed no significant neuronal loss in the CL→mPFC pathway after SAH. Therefore, the observed dysfunction of the CL→mPFC neural pathway is likely due to disrupted functional connectivity rather than cell death.

### SAH-induced myelin damage and axonal injury in the CL→mPFC neural pathway accompanied by oligodendrocyte dysfunction

**Fig. 3:**
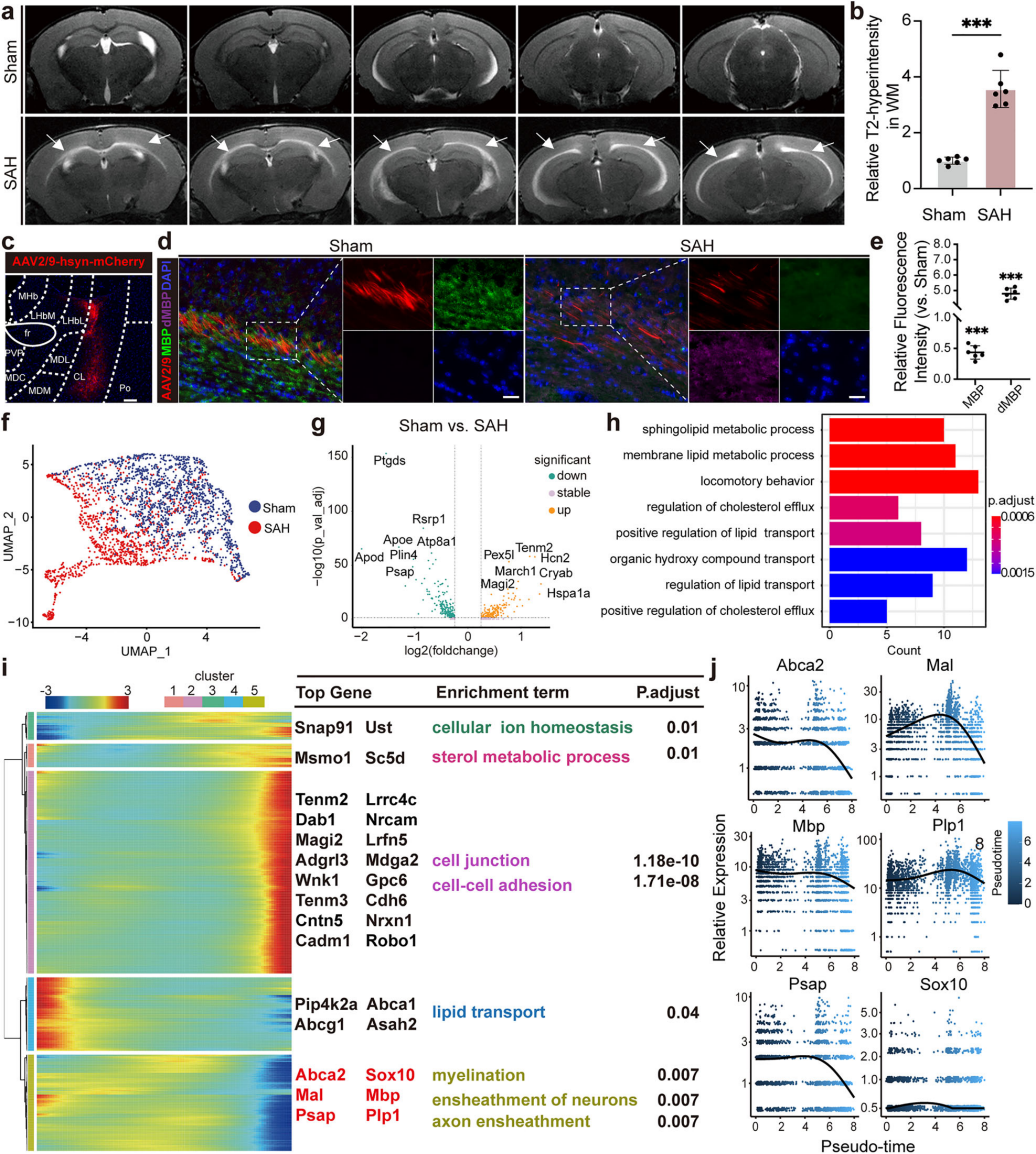
SAH induces myelin and axonal damage in the CL→mPFC pathway accompanied by oligodendrocyte dysfunction and downregulation of myelination-associated genes. **a** Representative T2-weighted MRI images. **b** Quantification of T2-hyperintensity in regions of interest (*n* = 6, ***P <0.001). **c** Representative image showing AAV2/9-hSyn-mCherry transfection, with mCherry (red) as the viral marker and 4′,6-diamidino-2-phenylindole (DAPI; blue) marking nuclei. Scale bar: 200 μm. **d** AAV2/9**-**hSyn-mCherry-labelled central lateral thalamus (CL) axons (red) in the corpus callosum co-stained for myelin basic protein (MBP; green) and degraded MBP (dMBP; Magenta) at 48 h post-subarachnoid haemorrhage (SAH). Scale bars: 50 µm. **e** Quantification of dMBP and MBP levels in mCherry-positive regions of the corpus callosum (*n* = 6, ***P <0.001, unpaired t-test). **f** Uniform manifold approximation and projection (UMAP) visualization of oligodendrocytes, showing distinct segregation between sham and SAH groups. **g** Volcano plot showing differential expression of genes in oligodendrocytes following SAH. **h** Pathway enrichment analysis of downregulated genes in oligodendrocytes, showing significant enrichment in lipid metabolism. **i** Gene Ontology term analysis of five gene clusters from pseudotime analysis, identifying enriched pathways related to myelination, lipid transport and ion homeostasis. **j** Downregulation of key myelination-related genes in pseudotime analysis, highlighting the significant downregulation of *Psap*, *Abca2*, *Mal* and *Mbp* following SAH. mPFC, medial prefrontal cortex.

The functional integrity of the central nervous system depends on highly organized neural pathways and efficient information transmission networks. As a critical signalling structure, white matter is composed of densely packed fibre bundles whose myelinated axons facilitate inter-regional communication and provide essential axonal protection^30^. Using a 9.4 T small-animal MRI scanner, we acquired T2-weighted images and observed T2 hyperintensities in several key white matter regions in SAH mice, including the corpus callosum, external capsule, fimbria, and internal capsule, cerebral peduncle and optic tract (Fig. 3a, b).To further investigate SAH-induced disruption of functional connectivity in the CL→mPFC pathway, we stereotaxically injected AAV2/9-hSyn-mCherry into the CL to trace CL-derived axons projecting to the corpus callosum, a representative white matter structure along this pathway (Fig. 3c, d). Two days post-surgery, immunofluorescence staining revealed increased accumulation of degraded myelin basic protein (dMBP) and reduced myelin basic protein (MBP) levels in mCherry-positive regions of the corpus callosum in SAH mice compared to sham controls (Fig. 3d, e), suggesting demyelination and myelin damage within the CL→mPFC pathway.

Oligodendrocytes are the principal cells responsible for generating and maintaining myelin sheaths, playing a vital role in supporting rapid signal conduction in the central nervous system^31,32^. Even after myelination, oligodendrocytes remain functionally active by sustaining axonal integrity through myelin channels^33^. To investigate the effects of SAH on oligodendrocyte function, we isolated oligodendrocytes from the snRNA-seq dataset. Re-clustering and dimensionality reduction revealed a clear segregation between sham and SAH groups, indicating marked alterations in oligodendrocyte cell states following SAH (Fig. 3f). Oligodendrocytes were predominantly linked to lipid metabolism (Fig. 3g, h). Given that lipids account for ~70% of myelin content and are essential for axonal insulation and signal transmission, the suppression of lipid metabolism suggests disrupted myelin homeostasis contributing to axonal injury.

To further characterize oligodendrocyte state transitions, we applied Monocle2 for single-nucleus trajectory analysis (Supplementary Fig. 5a, b). Cells from sham and SAH groups diverged after State 1 along the pseudotime trajectory. Gene expression dynamics identified five clusters with distinct transcriptional patterns, with Gene Ontology term enrichment revealing pathways related to ion homeostasis, sterol metabolism, lipid transport and myelination (Fig. 3i and Supplementary Fig. 5c–g). Notably, Cluster 5 — associated with myelination — exhibited progressive downregulation from sham to SAH. Key myelination-related genes within this cluster, including *Abca2*, *Sox10*, *Mal*, *Mbp*, *Psap* and *Plp1*, were among the most significantly downregulated, with *Abca2*, *Mal*, *Mbp* and *Psap* showing the largest reductions (Fig. 3j). *Psap*, a gene critical for myelin maintenance, showed the most pronounced downregulation in pseudotime analysis, suggesting a central role in SAH-induced myelin damage^34^.

### SAH-induced disruption of PSAP–GPR37 interaction in oligodendrocytes

**Fig. 4:**
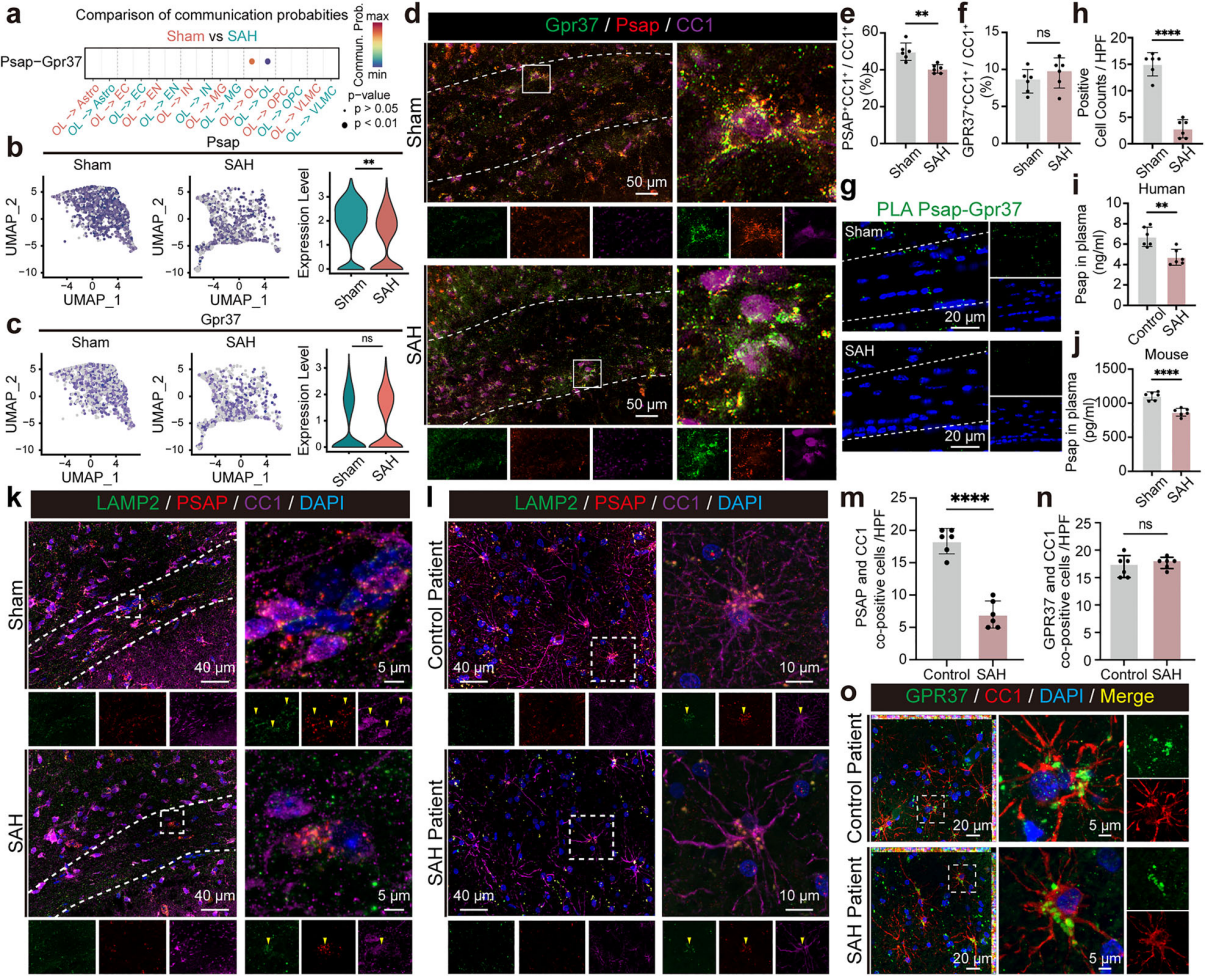
SAH reduces PSAP–GPR37 interactions via selective *Psap* downregulation in oligodendrocytes. Bubble plot showing changes in PSAP–GPR37 interactions following subarachnoid haemorrhage (SAH) (part **a**). Uniform manifold approximation and projection (UMAP) plot and violin plot showing *Psap* gene expression in sham and SAH mice (**P<0.01) (part **b**). (**c**) UMAP plot and violin plot showing *Gpr37* gene expression in sham and SAH mice (part **c**). Representative immunofluorescence images of GPR37 (green), PSAP (red) and CC1 (magenta) co-staining in the corpus callosum of sham and SAH mice (part **d**). Dashed lines outline the corpus callosum. Right panels show high-magnification images of the boxed regions. Scale bars: 50 μm. Quantification of the percentage of PSAP^+^CC1^+^ cells among total CC1^+^ cells in sham and SAH groups (*n* = 6, **P<0.01) (part **e**). Quantification of the percentage of GPR37^+^CC1^+^ cells among total CC1^+^ cells in sham and SAH groups (*n* = 6) (part **f**). Representative images of in situ proximity ligation assay (PLA) analysis showing PSAP and GPR37 interactions in the corpus callosum of brain tissue sections from sham and SAH mice (green represents interaction complexes, blue represents nuclei) (parts **g** and **h**; scale bar: 20 μm; *n* = 6, ****P<0.0001). ELISA-based quantification of PSAP levels in human and mouse plasma (*n* = 6, **P<0.01, ****P<0.0001) (parts **i** and **j**). Representative immunofluorescence images of LAMP2 (green), PSAP (red), CC1 (magenta) and 4′,6-diamidino-2-phenylindole (DAPI; blue) in the corpus callosum of sham and SAH mice (part **k**). Right panels show high-magnification images of the boxed regions. Yellow arrowheads indicate co-localization of PSAP with LAMP2-positive lysosomes in CC1-positive oligodendrocytes. Scale bars: 40 μm (left) and 5 μm (right). Representative immunofluorescence images of LAMP2 (green), PSAP (red), CC1 (magenta), and DAPI (blue) in brain tissue sections from human control individuals and patients with SAH (part **l**). Right panels show high-magnification images of the boxed regions. Yellow arrowheads indicate co-localization of PSAP with LAMP2^+^ lysosomes in CC1^+^ oligodendrocytes. Scale bars: 40 μm (left) and 10 μm (right). Quantification of PSAP^+^CC1^+^ cells per high-power field in tissue sections from human control individuals and patients with SAH (*n* = 6, ****P<0.0001) (part **m**). Quantification of GPR37^+^CC1^+^ cells per high-power field in tissue sections from human control individuals and patients with SAH (*n* = 6) (part **n**). Representative immunofluorescence images of GPR37 (green), CC1 (red), DAPI (blue) and merged channels in brain tissue sections from human control individuals and patients with SAH (part **o**). Right panels show high-magnification images of the boxed regions. Scale bars: 20 μm (left) and 5 μm (right). ns, not significant.

To investigate the mechanisms underlying myelin damage and oligodendrocyte dysfunction following SAH, we performed CellChat-based cell–cell interaction analysis. The results revealed a significant reduction in interaction strength in the SAH group compared with sham controls (Supplementary Fig. 6a, b). Notably, PSAP signalling pathways were markedly downregulated after SAH (Supplementary Fig. 6c). Ligand–receptor analysis confirmed this disruption, identifying PSAP as the ligand and GPR37 as the receptor, both expressed in oligodendrocytes (Fig. 4a). These findings are consistent with the observed reduction in oligodendrocyte self-interactions in SAH mice (Supplementary Fig. 6a).

To investigate the mechanism underlying the reduced PSAP–GPR37 interaction in oligodendrocytes following SAH, we examined *Psap* and *Gpr37* expression using snRNA-seq data. The analysis revealed that *Gpr37* was predominantly expressed in oligodendrocytes, with no significant difference in expression levels between the sham and SAH groups within this cell population (Fig. 4c and Supplementary Fig. 6g). Previous studies have reported that *Psap* is primarily expressed in neurons^35^. We further analysed *Psap* expression in our snRNA-seq data and found that, although *Psap* expression was significantly reduced in oligodendrocytes following SAH, neuronal *Psap* expression showed no significant difference between the two groups (Fig. 4b and Supplementary Fig. 6f). We also performed immunofluorescence co-staining for PSAP and the neuronal marker NeuN in the corpus callosum region. The staining results revealed that neurons were largely absent within the corpus callosum itself; however, neurons in the periventricular regions surrounding the corpus callosum indeed exhibited high PSAP expression (Supplementary Fig. 7a). To further validate these findings at the transcript level, we performed RNAscope FISH for *Psap* mRNA combined with CC1 and NeuN immunostaining. Consistent with the protein-level observations, abundant *Psap* mRNA puncta were detected in CC1-positive oligodendrocytes within the corpus callosum of sham mice, whereas *Psap* mRNA signals were markedly reduced following SAH (Supplementary Fig. 7b). Furthermore, Western blot analysis of the injured corpus callosum also indicated that PSAP protein levels were significantly decreased after SAH (Supplementary Fig. 7e).

Immunofluorescence staining further validated these findings: in mouse brain corpus callosum, the proportion of PSAP co-localized with CC1-positive mature oligodendrocytes was significantly decreased in the SAH group, while GPR37 and CC1 co-localization showed no significant difference between groups (Fig. 4d–f). Furthermore, Duolink proximity ligation assay (PLA) confirmed a significant reduction in PSAP–GPR37 interaction in the corpus callosum of SAH mice compared to sham controls, supporting the observed disruption (Fig. 4g, h). Given that PSAP is a secreted protein detectable in peripheral blood, we further examined plasma PSAP levels in both patients with SAH and in mice using ELISA to explore its potential as a non-invasive clinical biomarker for SAH-induced white matter injury. The results showed a consistent downregulation of plasma PSAP in both species (Fig. 4i, j), suggesting that circulating PSAP levels may reflect brain tissue changes and could serve as a candidate biomarker for monitoring white matter damage following SAH.

Co-staining of the lysosomal marker LAMP2 with PSAP revealed co-localization of PSAP and LAMP2 in oligodendrocytes from sham mice (yellow arrows), which was diminished in the SAH group (Fig. 4k). Similar observations were made in human brain tissue samples: brain tissue from control individuals showed prominent co-localization of PSAP with CC1-positive oligodendrocytes, whereas the number of PSAP and CC1 co-positive cells was significantly reduced in tissue from patients with SAH (Fig. 4l, m). Notably, the number of GPR37 and CC1 co-positive cells showed no significant difference between the two groups (Fig. 4n, o), suggesting that SAH primarily affects the expression and secretion of the ligand (PSAP) rather than those of the receptor (GPR37).

Additionally, we analysed the expression of *Grn*, which encodes progranulin — a protein known to interact with PSAP in regulating lysosomal function. In contrast to the significant downregulation of *Psap*, *Grn* expression in oligodendrocytes remained unchanged following SAH (Supplementary Fig. 6d, e), suggesting that the intracellular PSAP–progranulin pathway may be less affected in this context.

These findings suggest that the SAH-induced reduction in *Psap* expression may underlie the loss of PSAP–GPR37 interaction, potentially contributing to myelin damage and impaired functional connectivity in the CL→mPFC pathway.

### *Psap* overexpression enhances PSAP–GPR37 interactions and alleviates myelin and axonal damage following SAH

**Fig. 5:**
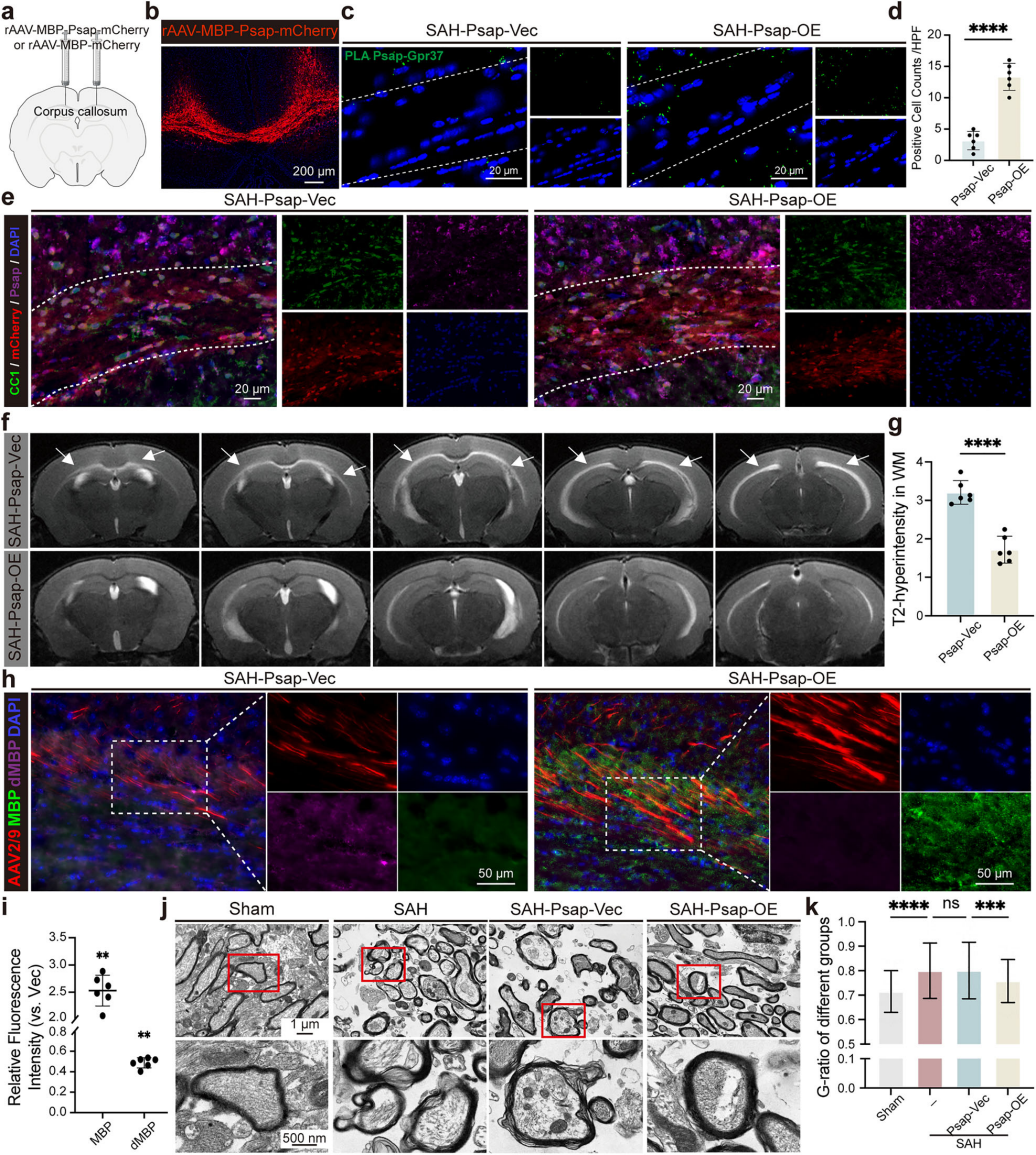
Oligodendrocyte-specific *Psap* overexpression enhances PSAP–GPR37 interactions and mitigates SAH-induced myelin and axonal damage in the CL→mPFC pathway. **a** Schematic representation of stereotactic injection of *Psap* overexpression virus into the corpus callosum. **b** Representative image showing *Psap* overexpression virus transfection, with mCherry (red) as the viral marker and 4′,6-diamidino-2-phenylindole (DAPI; blue) marking nuclei. Scale bar, 200 μm. **c** Representative in situ proximity ligation assay (PLA) images showing PSAP–GPR37 interactions in the corpus callosum of brain sections from subarachnoid haemorrhage (SAH) mice in the *Psap* overexpression (OE) and control (*Psap*-Vec) groups. Green signals represent interaction complexes, and blue signals represent nuclei. Scale bar, 20 μm. **d** The quantification of the PLA experiment representing the number of positive cells in each high-power field (*n* = 6, ****P<0.0001). **e** Representative immunofluorescence images of PSAP (magenta) and oligodendrocyte marker (CC1; green) staining in the corpus callosum of *Psap* OE and control (*Psap*-Vec) groups, with dashed lines outlining the corpus callosum. Scale bar: 20 μm. **f** Representative T2-weighted MRI images of the corpus callosum in *Psap* OE and control (*Psap*-Vec) groups following SAH. **g** Quantification of normalized T2 signal intensity in the corpus callosum of *Psap* OE and control (*Psap*-Vec) groups following SAH (*n* = 6, ****P<0.0001). **h** AAV2/9-hSyn-mCherry-labelled central lateral thalamus (CL) axons (red) in the corpus callosum co-stained for myelin basic protein (MBP) (green) and degraded MBP (dMBP; magenta). Scale bars: 50 µm. **i** Quantification of dMBP and MBP levels in mCherry^+^ regions of the corpus callosum (OE/Vec, *n* = 6, **P < 0.01, unpaired *t*-test). **j** Representative electron microscopy images of myelin in the corpus callosum from different groups. Scale bars: 1 μm (top panels) and 500 nm (bottom panels). **k** Quantification of g-ratio from myelin ultrastructure shown in part **i**. *n* >100, ***P < 0.001, ****P < 0.0001. mPFC, medial prefrontal cortex; ns, not significant.

To determine whether modulating *Psap* expression could attenuate myelin and axonal damage after SAH, we overexpressed *Psap* specifically in oligodendrocytes using an AAV vector driven by the MBP promoter and carrying an mCherry tag. The virus was bilaterally injected into the corpus callosum via stereotaxic surgery (Fig. 5a), and strong mCherry fluorescence confirmed successful transfection (Fig. 5b). To confirm the cell-type specificity of viral-mediated *Psap* overexpression, we performed immunofluorescence staining with multiple cellular markers. The results showed that the vast majority of mCherry-positive cells (indicating viral transduction) co-localized with CC1-positive cells, whereas no obvious co-localization was observed with neurons, microglia or astrocytes (Supplementary Fig. 7c, d), confirming that AAV-mediated *Psap* overexpression specifically targeted oligodendrocytes. Immunofluorescence staining further demonstrated significant upregulation of *Psap* in oligodendrocytes in the SAH+*Psap* overexpression group (Fig. 5e). Additionally, PLA revealed enhanced PSAP–GPR37 interactions in the corpus callosum following *Psap* overexpression (Fig. 5c, d). To evaluate the impact of *Psap* overexpression on white matter integrity, T2-weighted MRI was conducted, revealing significantly reduced T2 hyperintensities in the corpus callosum of the SAH+*Psap* overexpression group compared to the SAH+vector (Vec) group (Fig. 5f, g). In addition, AAV2/9-hSyn-mCherry was stereotaxically injected into the CL to trace CL-derived axons projecting to the corpus callosum (Fig. 3e). Immunofluorescence analysis showed that *Psap* overexpression increased MBP fluorescence and reduced dMBP fluorescence in mCherry-positive regions compared to the SAH+Vec group, indicating preserved myelin structure in the CL→mPFC pathway (Fig. 5h, i). Transmission electron microscopy further confirmed that *Psap* overexpression mitigated SAH-induced myelin damage, as evidenced by more intact myelin sheath structures in the SAH+*Psap* overexpression group (Fig. 5j). Quantitative analysis of the g-ratio showed a significant decrease in the SAH+*Psap* overexpression group, reflecting thicker myelin sheaths and improved myelin preservation relative to the SAH+Vec group (Fig. 5k). Together, these findings provide compelling evidence that *Psap* overexpression enhanced PSAP–GPR37 interactions, preserved myelin integrity and reduced axonal injury following SAH, likely through a neuroprotective effect on oligodendrocytes. This protection is associated with preserved corpus callosum integrity, highlighting a critical role for PSAP–GPR37 signalling in maintaining functional neural connectivity after SAH.

### *Psap* overexpression improves CL→mPFC neural pathway functional connectivity and alleviates consciousness impairments following SAH

**Fig. 6:**
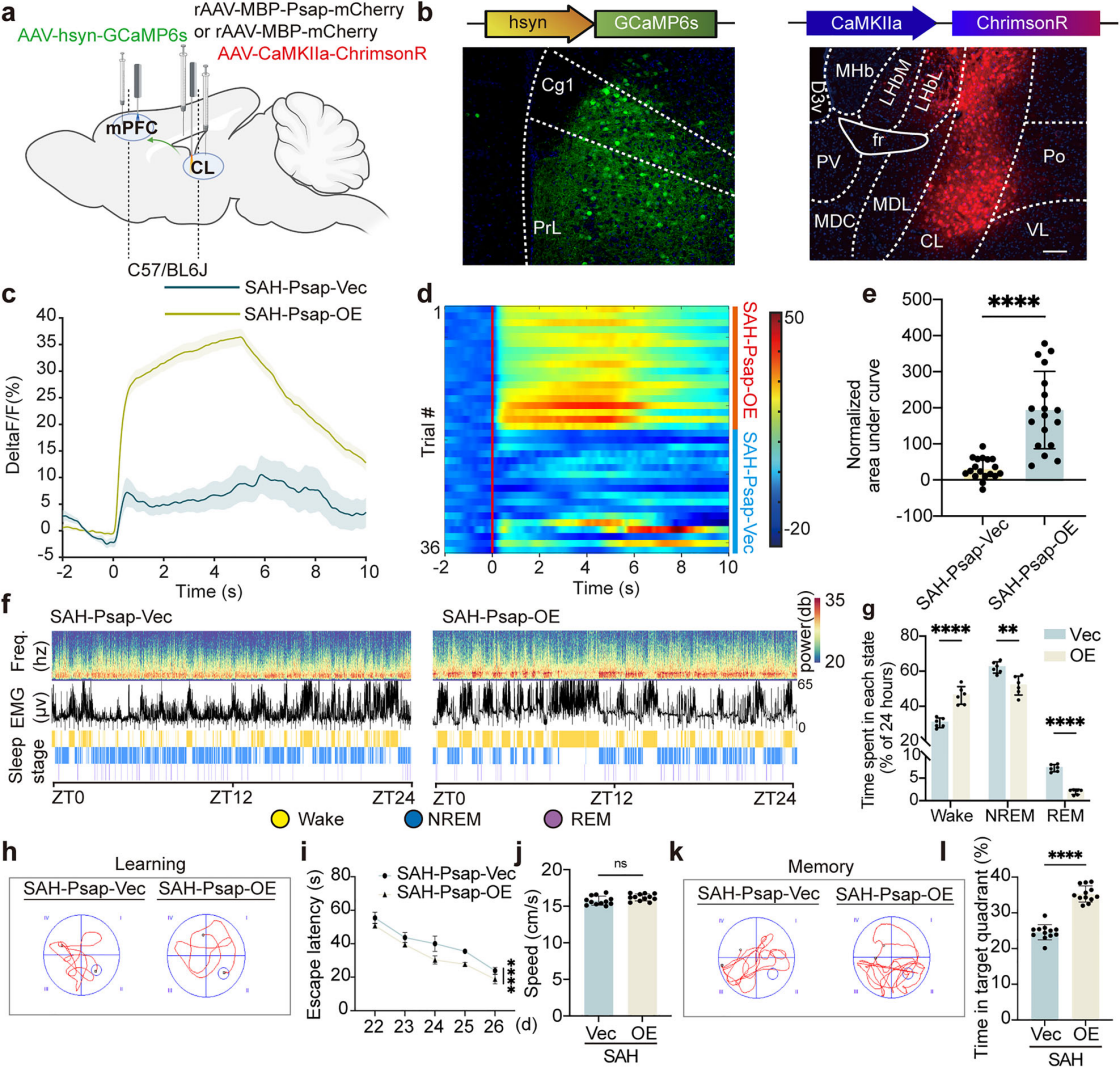
*Psap* overexpression restores CL→mPFC functional connectivity and improves consciousness and cognitive impairments following subarachnoid haemorrhage. Schematic of the optogenetic experimental design (part **a**). An adeno-associated viral (AAV) vector encoding ChrimsonR was injected into the central lateral thalamus (CL), an AAV vector encoding GCaMP6s into the medial prefrontal cortex (mPFC), and a *Psap*-overexpressing AAV vector was injected into the corpus callosum. Representative images showing successful transfection of the light-sensitive protein ChrimsonR and the calcium indicator GCaMP6s in the CL and mPFC, respectively (part **b**). Post-stimulation average histogram of ΔF/F calcium signals aligned across the control (Vec) and *Psap* overexpression (OE) groups, with the black line indicating the mean and shaded area representing the standard error of the mean (part **c**; scale bar: 100 μm). Heatmap of ΔF/F calcium signals in the control (Vec) and *Psap* OE groups, with the colour bar indicating individual mice and groupings (part **d**). Quantification of normalized calcium signal area under the curve in the control (Vec) and *Psap* OE groups (*n* = 6, ****P <0.0001) (part **e**). Representative electroencephalogram (EEG) and electromyogram (EMG) recordings, and sleep–wake stage analysis over 24 h, recorded 48 h post-surgery in the control and *Psap* OE groups (part **f**). Proportion of time spent in each sleep–wake stage in the control (Vec) and *Psap* OE groups (*n* = 6, **P <0.01, ****P <0.0001) (part **g**). Representative images from the Morris water maze and probe tests in the control and *Psap* OE groups (parts **h** and **k**). Quantitative analysis of escape latency, swimming speed and time spent in the target quadrant during the Morris water maze and probe tests in the control and *Psap* OE groups (*n* = 12, ****P <0.0001, two-way ANOVA for part **i**, unpaired *t*-test for parts **j** and **l**) (parts **i**, **j** and **l**). ns, not significant.

To assess the effect of *Psap* overexpression on CL→mPFC functional connectivity, optogenetic experiments were performed. Channelrhodopsin ChrimsonR was stereotaxically injected into the CL, GCaMP6s into the mPFC, and *Psap*-overexpressing AAV bilaterally into the corpus callosum (Fig. 6a, b). After 21 days of viral expression, SAH modelling was performed, followed by optogenetic stimulation 48 h later. Upon 589 nm light stimulation of the CL, the SAH+*Psap* overexpression group showed significantly enhanced calcium signals in the downstream mPFC compared to the SAH+Vec group (Fig. 6c–e), indicating restored functional connectivity in the CL→mPFC pathway.

To assess whether *Psap* overexpression improved consciousness, EEG and EMG recordings and sleep–wake analysis were performed 24 h post-SAH (Fig. 6f, g). Compared with the SAH+Vec group, mice in the *Psap* overexpression group exhibited a significantly higher proportion of wakefulness and reduced proportions of NREM and REM sleep, suggesting improved consciousness after SAH.

Consciousness impairments induced by brain injury are known to affect cognitive recovery^36^. The CL→mPFC neural pathway plays a crucial role in higher-order cognitive functions^3,37^. Therefore, we further assessed spatial learning and memory using the Morris water maze. During the acquisition phase, SAH mice showed significantly longer escape latencies compared to sham controls. Notably, the SAH+*Psap* overexpression group demonstrated a significant reduction in escape latency compared to the SAH+Vec group, particularly on days 23–26, suggesting improved spatial learning. In the probe trial, the SAH+*Psap* overexpression group also exhibited more platform crossings and spent more time in the target quadrant relative to the SAH+Vec group (Fig. 6h–l), indicating improved cognitive performance following *Psap* overexpression.

### HuPSAP restores CL→mPFC functional connectivity and alleviates consciousness and cognitive impairments following SAH

**Fig. 7:**
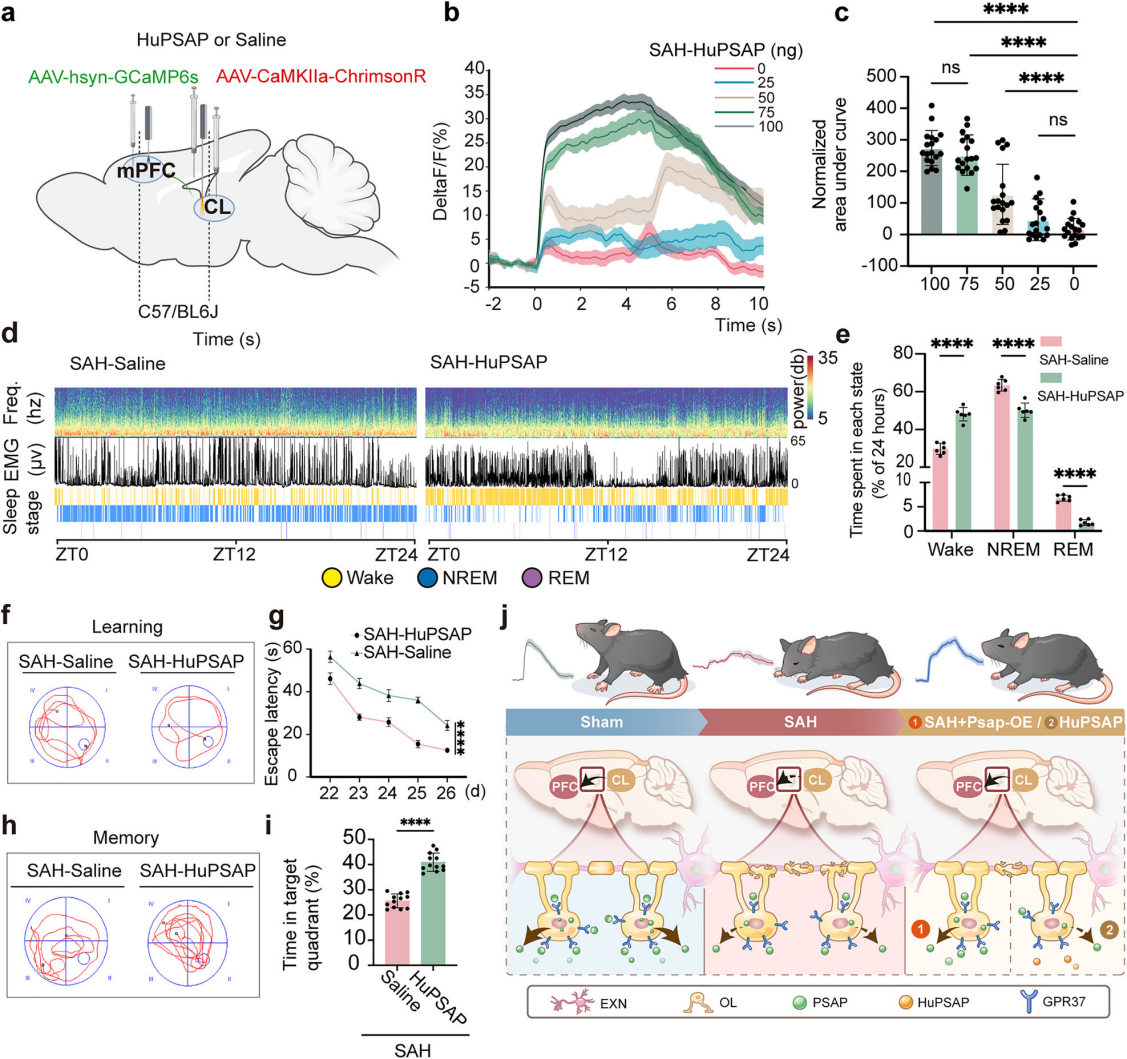
HuPSAP restores CL→mPFC functional connectivity and ameliorates consciousness and cognitive impairments in SAH mice. Schematic of the optogenetic experiment (part **a**). An adeno-associated viral vector (AAV) encoding ChrimsonR was injected into the central lateral thalamus (CL), an AAV vector encoding GCaMP6s was injected into the medial prefrontal cortex (mPFC) and recombinant human PSAP (HuPSAP) was stereotactically delivered into the corpus callosum. Post-stimulation average histogram of ΔF/F calcium signals across HuPSAP concentration groups, with the thick black line representing the mean and the shaded area representing the standard error of the mean (part **b**). Quantification of normalized calcium signal area under the curve in different HuPSAP concentration groups (*n* = 6, ****P <0.0001, one-way ANOVA) (part **c**). Representative electroencephalogram (EEG) and electromyogram (EMG) recordings and sleep–wake staging over 24 h in HuPSAP and saline control groups (part **d**). Proportion of time spent in each sleep–wake stage in the HuPSAP and saline control groups (*n* = 6, ****P <0.0001) (part **e**). Representative images from the Morris water maze and probe tests in HuPSAP and saline control groups (parts **f** and **h**). Quantitative analysis of escape latency and time spent in the target quadrant during the Morris water maze and probe tests (*n* = 12, ****P <0.0001, two-way ANOVA for part **g**, unpaired t-test for part **i**) (parts **g** and **i**). Subarachnoid haemorrhage (SAH) induces consciousness impairment and downregulates *Psap* expression in oligodendrocytes, disrupting PSAP–GPR37 interactions that are essential for maintaining white matter integrity (part **j**). Targeted *Psap* overexpression in oligodendrocytes or administration of HuPSAP restores PSAP–GPR37 signalling, attenuates SAH-induced white matter damage, and reestablishes CL→mPFC functional connectivity, thereby improving consciousness. These findings identify PSAP as a potential therapeutic target for SAH-induced disorders of consciousness. EXN, excitatory neurons; ns, not significant; OL, oligodendrocytes.

We next investigated the therapeutic effects of HuPSAP on CL→mPFC neural pathway impairment following SAH using optogenetic techniques. ChrimsonR was stereotactically injected into the CL and GCaMP6s into the mPFC. HuPSAP was injected into the corpus callosum at concentrations of 25 ng, 50 ng, 75 ng and 100 ng. Following a 3-day recovery period, SAH was induced, and optogenetic stimulation was performed 48 h post-SAH (Fig. 7a and Supplementary Fig. 8a). In response to 589 nm light stimulation of the upstream CL nucleus, the HuPSAP-treated group, particularly at the 75 ng concentration, exhibited significantly enhanced calcium signalling in the downstream mPFC region compared to the SAH+saline group, indicating improved CL→mPFC neural pathway functional connectivity (Fig. 7b, c and Supplementary Fig. 8b). No significant difference was observed between the 75 ng and 100 ng HuPSAP groups.

To further evaluate the effect of HuPSAP on SAH-induced white matter injury, AAV2/9-hSyn-mCherry was stereotactically injected into the CL to anterogradely label axons traversing the corpus callosum. Immunofluorescence analysis showed a marked reduction in dMBP accumulation in mCherry-positive regions in HuPSAP-treated mice compared to saline controls, indicating attenuated demyelination (Supplementary Fig. 8c, d).

To assess whether HuPSAP injection also alleviated consciousness and cognitive impairments, EEG and EMG recordings and sleep–wake analysis were conducted 24 h post-SAH. HuPSAP-treated SAH mice exhibited significantly increased wakefulness and decreased NREM and REM sleep compared to saline-treated controls, indicating improved consciousness (Fig. 7d, e). Cognitive function was further evaluated using the Morris water maze. During the acquisition phase, swimming speed did not differ significantly between groups. However, HuPSAP-treated mice exhibited significantly reduced escape latency compared to saline-treated controls, particularly on days 23–26, indicating enhanced spatial learning (Fig. 7f, g and Supplementary Fig. 8e). In the probe trial, HuPSAP-treated mice crossed the platform more frequently and spent more time in the target quadrant, further supporting a cognitive benefit of HuPSAP following SAH (Fig. 7h, i).

### Oligodendrocyte-specific *Gpr37* knockdown attenuates the therapeutic effects of HuPSAP

Given that PSAP is a secreted protein, we sought to determine whether the therapeutic effects of HuPSAP are mediated through oligodendrocyte GPR37. To address this question, we employed PLP-CreERT2 transgenic mice to achieve oligodendrocyte-specific *Gpr37* knockdown. Cre-dependent AAV vectors carrying either *Gpr37* short hairpin RNA (shRNA) or scramble control shRNA were bilaterally injected into the corpus callosum (Supplementary Fig. 9a). Immunofluorescence staining confirmed that viral transduction (mCherry positivity) was restricted to CC1-positive oligodendrocytes and did not occur in NeuN-positive neurons (Supplementary Fig. 9b). Western blot analysis demonstrated that GPR37 protein levels were significantly reduced in the SAH+shRNA(*Gpr37*) group compared to SAH+shRNA(scramble) controls (Supplementary Fig. 9c). This knockdown efficiency was further validated by immunofluorescence staining, which showed markedly reduced GPR37 immunoreactivity in mCherry-positive cells (Supplementary Fig. 9d).

To determine whether oligodendrocyte GPR37 is required for HuPSAP’s therapeutic effects, we administered HuPSAP to SAH mice with or without oligodendrocyte-specific *Gpr37* knockdown. EEG and EMG recordings and sleep–wake analysis revealed that the beneficial effects of HuPSAP on consciousness were significantly attenuated when oligodendrocyte *Gpr37* was knocked down. Specifically, SAH-HuPSAP+shRNA(*Gpr37*) mice exhibited reduced wakefulness, increased NREM sleep and decreased REM sleep compared with SAH-HuPSAP+shRNA(scramble) controls (Supplementary Fig. 9e, f). These findings demonstrate that the therapeutic effects of secreted PSAP are primarily mediated through oligodendrocyte GPR37, supporting the critical role of PSAP–GPR37 signalling in maintaining consciousness following SAH.

### Oligodendrocyte-specific *Psap*-knockdown recapitulates consciousness impairment in sham mice

The above experiments demonstrated that *Psap* overexpression rescues SAH-induced consciousness deficits. We next asked the converse question: whether the loss of endogenous oligodendrocyte PSAP alone is sufficient to impair consciousness. We performed oligodendrocyte-specific*Psap*-knockdown in sham mice using MBP promoter-driven AAV-shRNA(*Psap*) bilaterally injected into the corpus callosum (Supplementary Fig. 10a). Oligodendrocyte specificity and knockdown efficiency were validated by immunofluorescence and Western blot, respectively (Supplementary Fig. 10b–d). Notably, PSAP protein levels in the shRNA(*Psap*) group were reduced to levels comparable to those observed in SAH mice (Supplementary Fig. 10c). EEG and EMG recordings revealed that *Psap*-knockdown mice exhibited significantly reduced wakefulness and increased NREM and REM sleep compared to scramble controls (Supplementary Fig. 10e, f). Although the magnitude of these changes was milder than that observed after SAH (Fig. 1c), the direction and pattern of the sleep–wake alterations were consistent, indicating that oligodendrocyte PSAP loss alone is sufficient to impair consciousness.

## Discussion

In this study, we investigated the mechanisms underlying consciousness impairment following SAH, focusing on the disruption of the CL→mPFC neural pathway and its potential therapeutic modulation by PSAP. Our findings indicate that SAH leads to severe consciousness disturbances in mice, characterized by reduced wakefulness and altered sleep patterns. Optogenetic analyses revealed impaired functional connectivity within the CL→mPFC neural pathway following SAH, which was associated with significant white matter damage, particularly in the corpus callosum. Notably, PSAP, a protein crucial for myelin integrity and oligodendrocyte function, was significantly downregulated in oligodendrocytes following SAH. Overexpression of PSAP or HuPSAP treatment alleviated the white matter damage, restored CL→mPFC neural pathway functional connectivity, and significantly improved both consciousness and cognitive function in SAH mice. These findings underscore the critical role of PSAP–GPR37 interactions in maintaining neural pathway integrity and suggest that PSAP may serve as a promising therapeutic target for the treatment of SAH-induced consciousness disturbances (Fig. 7j).

SAH is a severe cerebrovascular condition frequently associated with significant consciousness disturbances, ranging from lethargy to coma^38^. Epidemiological studies indicate that up to 60% of patients with SAH experience impaired consciousness during the acute phase, which is strongly associated with poor clinical outcomes, including higher mortality and long-term neurological deficits^1^. While many studies have focused on neurological deficits, such as spatial memory and cognitive impairments in SAH mouse models^39,40^, research on consciousness disturbances following SAH in these models remains limited. EEG and EMG analyses are crucial tools to study consciousness impairments and sleep–wake states^41–43^. A retrospective study of patients with acute SAH who underwent continuous EEG monitoring for consciousness impairment identified early corticothalamic connectivity disruption, which correlated with functional outcomes and in-hospital mortality, highlighting its potential as a predictive tool pending prospective validation^44^. In our study, we conducted EEG-based and EMG-based sleep–wake analysis on a SAH mouse model and observed a significant reduction in wakefulness across the light–dark cycle, with a frequency of wakefulness impairment at 58%, which closely aligns with the incidence of consciousness disturbances observed in patients with SAH^1^. These findings offer valuable insights and may serve as a helpful experimental foundation for the further investigation of SAH-induced consciousness disturbances and associated neurological deficits in mouse models.

Previous studies on the mechanisms of consciousness impairment following SAH have primarily focused on global brain injury factors such as elevated intracranial pressure, reduced cerebral perfusion, and widespread cortical or subcortical damage^45,46^. However, a substantial body of research has emphasized the critical role of thalamocortical neural pathways in consciousness regulation, particularly the involvement of the CL→mPFC neural pathway in maintaining wakefulness, attention and cognitive functions^3,37^. Specifically, the CL serves as a hub for the integration of sensory and motor inputs, relaying these signals to the mPFC and other cortical areas, while the mPFC plays a central role in executive functions, decision-making and maintaining wakefulness^5,47^. In the context of consciousness disorders, deep brain stimulation targeting the CL has been shown to promote wakefulness and assist in recovery from coma in patients with severe brain injuries, further highlighting the importance of the CL→mPFC neural pathway in consciousness regulation under pathological conditions^47,48^. However, the role of the CL→mPFC neural pathway in consciousness disturbances following SAH remains underexplored. In our study, we observed impaired functional connectivity within the CL→mPFC neural pathway after SAH, which was closely associated with reduced wakefulness in SAH mice.

Stroke disrupts functional connectivity within brain networks, often attributed to cortical damage. However, data from 114 patients with stroke suggest that structural disconnections in white matter pathways more accurately account for these disruptions than focal cortical lesions^9^. Moreover, studies have shown that indirect estimates of structural connectivity damage can reliably predict post-stroke behavioural deficits, with accuracy comparable to lesion-based assessments^49^. Similarly, SAH induces white matter damage, including axonal injury and myelin degradation, primarily in the corpus callosum, which severely disrupts neural signal functional connectivity^50,51^. This damage impairs connectivity in neural pathways, including thalamocortical and corticocortical pathways^31,32^, highlighting the importance of elucidating how white matter injury contributes to SAH-induced consciousness impairment. Using a combination of optogenetics and snRNA-seq, we found that SAH did not cause severe neuronal loss in the CL region. Instead, it triggered oligodendrocyte dysfunction, which likely contributes to white matter damage and disruption of CL→mPFC pathway integrity.

Oligodendrocytes, which generate and maintain myelin sheaths in the central nervous system, are essential for proper neural pathway function^11^. Damage to oligodendrocytes and subsequent myelin degeneration are significant contributors to cognitive deficits in various neurological conditions, including stroke, multiple sclerosis and traumatic brain injury^11^. However, the specific mechanisms underlying oligodendrocyte dysfunction in the context of SAH remain poorly understood. Our study provides new insights by demonstrating that PSAP, a key protein required for oligodendrocyte function, is downregulated following SAH. These findings underscore the essential role of oligodendrocyte integrity in maintaining neural connectivity and support PSAP as a promising therapeutic target to improve consciousness and neurological outcomes following SAH.

PSAP is a multifunctional protein that supports glial function and acts as a neurotrophic factor through interactions with G protein-coupled receptors such as GPR37 and GPR37L1 (refs. ^14,35,52–54^). Studies have shown that PSAP plays a protective role in maintaining myelin integrity and preventing oligodendrocyte loss under pathological conditions^54,55^. Reduced PSAP expression has been associated with white matter damage, while PSAP upregulation mitigates both myelin and axonal damage^13–15^. Single-cell analysis of spinal cord injury has further highlighted the importance of PSAP–GPR37L1 interactions in oligodendrocyte lineage cells^56^. However, the role of PSAP in SAH-induced white matter damage and neural pathway dysfunction remains poorly understood.

In the present study, using snRNA-seq and proximity ligation assays, we found that PSAP expression in oligodendrocytes was significantly reduced following SAH, accompanied by diminished PSAP–GPR37 interactions. This finding was further corroborated at the transcript level by RNAscope FISH, which revealed a marked reduction in *Psap* mRNA puncta within CC1-positive oligodendrocytes after SAH. Importantly, a corresponding decrease in plasma PSAP levels was detected in both SAH mice and human patients. Since blood-based biomarkers offer significant clinical advantages, including non-invasive sampling, ease of serial monitoring and applicability to large-scale screening, our findings suggest that plasma PSAP may serve as a potential biomarker for assessing SAH-related white matter injury and predicting consciousness outcomes, although future prospective studies are warranted to validate its diagnostic and prognostic value. Critically, our study establishes a comprehensive causal framework for the role of oligodendrocyte PSAP–GPR37 signalling in consciousness regulation through convergent gain-of-function and loss-of-function evidence. On the one hand, oligodendrocyte-specific *Psap* overexpression restored PSAP–GPR37 interactions, protected myelin integrity and rescued consciousness deficits after SAH. On the other hand, oligodendrocyte-specific *Psap* knockdown in sham mice was sufficient to induce consciousness impairment in the absence of haemorrhagic injury, establishing a direct causal link between oligodendrocyte PSAP loss and consciousness disturbances. The milder phenotype observed in the knockdown model compared to SAH is consistent with the multifactorial nature of SAH pathology, in which *Psap* downregulation represents one critical, but not the sole, contributing mechanism. Complementing these findings, oligodendrocyte-specific *Gpr37* knockdown abolished the therapeutic effects of exogenous HuPSAP, confirming receptor dependency. Together, these results — sufficiency (*Psap* knockdown), rescue (*Psap* overexpression) and receptor dependency (*Gpr37* knockdown) — converge to demonstrate that endogenous oligodendrocyte PSAP, acting through GPR37, is a major driver of consciousness regulation following SAH. Building on these mechanistic insights, we further demonstrated that exogenous HuPSAP treatment significantly alleviated myelin damage, restored functional connectivity in the CL→mPFC neural pathway, and improved both consciousness and cognitive function after SAH. These findings not only reinforce the central role of PSAP in white matter protection but also highlight HuPSAP as a promising therapeutic candidate to address consciousness and cognitive impairments following SAH.

An important consideration in our study is the secretory nature of PSAP. As a secreted protein, PSAP could potentially act on multiple cell types expressing its receptor GPR37. Our snRNA-seq data revealed that *Gpr37* is predominantly expressed in oligodendrocytes, suggesting that oligodendrocytes are the primary target cells for secreted PSAP. To directly test this hypothesis, we performed oligodendrocyte-specific *Gpr37* knockdown experiments using PLP-CreERT2 mice. Strikingly, selective knockdown of *Gpr37* in oligodendrocytes significantly attenuated the therapeutic effects of exogenous HuPSAP administration, demonstrating that the beneficial effects of secreted PSAP are largely mediated through oligodendrocyte GPR37 signalling. It is worth noting that our findings appear to complement, rather than contradict, a recent study suggesting that GPR37 negatively regulates oligodendrocyte differentiation during development^57^. The context-dependent roles of GPR37 signalling — inhibiting differentiation during development versus promoting survival and function during pathological injury — likely reflect distinct downstream signalling cascades activated under different physiological conditions. Our data specifically demonstrate that, in the context of SAH-induced injury, PSAP–GPR37 signalling plays a protective role in maintaining oligodendrocyte function and myelin integrity.

Our study focused on oligodendrocyte-derived PSAP based on our finding that PSAP downregulation following SAH occurs predominantly in oligodendrocytes. While our data demonstrate that oligodendrocyte-specific GPR37 is required for the therapeutic effects of secreted PSAP, we did not directly compare the efficacy of PSAP overexpression in different cell types (for example, neurons versus oligodendrocytes). Future studies employing cell-type-specific PSAP overexpression using neuron-specific promoters would provide additional insight into whether the cellular source of secreted PSAP influences its signalling specificity and therapeutic potential in white matter pathology.

Astrocytes have attracted considerable attention for their involvement in white matter injury^58^. Neuroinflammatory reactive astrocytes are implicated in perinatal white matter injury^20^. Additionally, in chronic cerebral hypoperfusion, the interaction between astrocytes and oligodendrocyte lineage cells plays a crucial role in maintaining white matter homeostasis^21^. Previous studies have shown that PSAP protects astrocytes from oxidative stress via endogenous astrocytic GPR37 or GPR37L1 (ref. ^14^). In the present study, we primarily focused on the role of oligodendrocyte PSAP–GPR37 signalling in SAH-induced white matter injury and consciousness impairment. However, since PSAP is a secreted protein, it remains unclear whether AAV-mediated overexpression of *Psap* in oligodendrocytes, or stereotactic injection of recombinant HuPSAP, may affect non-oligodendrocyte brain cells, particularly astrocytes. Moreover, *Psap* has been reported to promote the expression of myelin-associated proteins^54^. Whether AAV-mediated *Psap* overexpression or HuPSAP delivery modulates myelin protein expression, thereby influencing white matter integrity and consciousness outcomes after SAH, warrants further investigation.

It is noteworthy that PSAP exerts its biological functions through two distinct mechanisms: an intracellular pathway involving lysosomal sphingolipid metabolism in conjunction with progranulin (PGRN), and an extracellular pathway mediated by secreted PSAP binding to cell surface receptors GPR37 and GPR37L1. Published studies have demonstrated that PGRN serves as a molecular chaperone for PSAP, facilitating its lysosomal delivery and subsequent generation of saposins essential for lipid degradation^59–61^. In our study, snRNA-seq analysis revealed that *Grn* expression in oligodendrocytes was not significantly altered following SAH, whereas *Psap* was markedly downregulated. This differential expression pattern, combined with reduced plasma PSAP levels and diminished PSAP–GPR37 interactions observed in both mice and human patients, suggests that SAH predominantly disrupts the extracellular neurotrophic function of PSAP rather than its intracellular lysosomal pathway. Nevertheless, the potential contribution of PSAP–PGRN interactions to lysosomal dysfunction and sphingolipid accumulation in oligodendrocytes following SAH warrants further investigation, as subtle alterations in this pathway could still influence myelin homeostasis independently of transcriptional changes in *Grn*.

Despite promising results, our study has several limitations. First, while the animal model used is effective for investigating the pathophysiology of SAH, it may not fully replicate the complexity of human SAH. Additionally, while our *Psap*-knockdown experiments demonstrate sufficiency in sham mice, the relative contribution of oligodendrocyte *Psap* loss versus other pathological mechanisms (for example, neuroinflammation, vasospasm) to consciousness impairment during SAH remains to be quantitatively delineated in future studies. Therefore, further validation in larger, more clinically relevant models, such as in non-human primates, is necessary to assess the translatability of our findings. Second, while *Psap* overexpression enhances PSAP–GPR37 interactions and protects myelin, the specific downstream signalling pathways remain unclear, necessitating further mechanistic studies to elucidate how PSAP modulates oligodendrocyte function and myelin integrity, particularly in the context of neuroinflammation and glial cell interactions. Third, the long-term safety and efficacy of PSAP-based therapies, including recombinant HuPSAP, were not evaluated, highlighting the need for future preclinical trials to assess sustained effects and potential side effects. To address these limitations, future research should refine experimental models to better mimic the human condition, particularly by using species with similar white matter structures and cognitive functions. Additionally, deeper investigation is required to identify the specific signalling pathways involved in the neuroprotective effects of PSAP, including exploring interactions with other key proteins involved in myelin metabolism and oligodendrocyte differentiation. Long-term effects of PSAP overexpression and HuPSAP administration in diverse animal models, including those with chronic SAH or comorbid conditions, will be crucial to refining PSAP as a therapeutic target in clinical settings.

In summary, our study significantly advances the understanding of how white matter damage and oligodendrocyte dysfunction contribute to consciousness and cognitive deficits following SAH. By highlighting the role of PSAP in maintaining myelin integrity and restoring CL→mPFC neural pathway function, our findings provide new therapeutic insights that could be translated into clinical strategies to treat SAH-induced neurological impairments. Our results also open new avenues to explore the potential of PSAP-based therapies in other conditions characterized by white matter damage.

Although this study demonstrates the neuroprotective role of the PSAP–GPR37 axis in SAH-induced consciousness disorders, several limitations should be acknowledged. First, the rodent model may not fully recapitulate the complexity of the human brain, including differences in myelin composition and common comorbidities such as hypertension. Second, the downstream signalling mechanisms of PSAP–GPR37 remain incompletely characterized. Third, therapeutic efficacy was assessed only during the acute phase, without long-term evaluation of functional recovery or safety. Future studies employing large-animal models, advanced imaging techniques and molecular tracing will be essential to validate translational relevance and refine delivery strategies.

## Supplementary information

Supplementary Information

Dateset1

## Data Availability

Single-nucleus RNA sequencing data have been deposited at GEO and are publicly available as of the date of publication. Accession number is: GSE295805. All data reported in this paper will be shared by the lead contact upon request.

## Code availability

All original code has been deposited at Zenodo at 10.5281/zenodo.15275572 and is publicly available as of the date of publication. Any additional information required to reanalyse the data reported in this paper is available from the lead contact upon request.

## References

[1] Suwatcharangkoon, S. et al. Loss of consciousness at onset of subarachnoid hemorrhage as an important marker of early brain injury. *JAMA Neurol.* **73**, 28–35 (2016). 26552033 10.1001/jamaneurol.2015.3188

[2] Helbok, R. et al. Cerebral tau is elevated after aneurysmal subarachnoid haemorrhage and associated with brain metabolic distress and poor functional and cognitive long-term outcome. *J. Neurol. Neurosurg. Psychiatry* **86**, 79–86 (2015). 24741064 10.1136/jnnp-2013-307326

[3] Redinbaugh, M. J. et al. Thalamus modulates consciousness via layer-specific control of cortex. *Neuron* **106**, 66–75.e12 (2020). 32053769 10.1016/j.neuron.2020.01.005 PMC7243351

[4] Luppi, A. I. et al. Consciousness-specific dynamic interactions of brain integration and functional diversity. *Nat. Commun.* **10**, 4616 (2019). 31601811 10.1038/s41467-019-12658-9 PMC6787094

[5] Edlow, B. L., Claassen, J., Schiff, N. D. & Greer, D. M. Recovery from disorders of consciousness: mechanisms, prognosis and emerging therapies. *Nat. Rev. Neurol.* **17**, 135–156 (2021). 33318675 10.1038/s41582-020-00428-x PMC7734616

[6] Panagiotaropoulos, T. I. An integrative view of the role of prefrontal cortex in consciousness. *Neuron* **112**, 1626–1641 (2024). 38754374 10.1016/j.neuron.2024.04.028

[7] Huo, Y., Chen, H. & Guo, Z. V. Mapping functional connectivity from the dorsal cortex to the thalamus. *Neuron* **107**, 1080–1094.e5 (2020). 32702287 10.1016/j.neuron.2020.06.038

[8] Sanchez, E. et al. Brain white matter damage and its association with neuronal synchrony during sleep. *Brain* **142**, 674–687 (2019). 30698667 10.1093/brain/awy348 PMC6391600

[9] Griffis, J. C., Metcalf, N. V., Corbetta, M. & Shulman, G. L. Structural disconnections explain brain network dysfunction after stroke. *Cell Rep.* **28**, 2527–2540.e9 (2019). 31484066 10.1016/j.celrep.2019.07.100 PMC7032047

[10] Reijmer, Y. D. et al. Microstructural white matter abnormalities and cognitive impairment after aneurysmal subarachnoid hemorrhage. *Stroke* **49**, 2040–2045 (2018). 30354997 10.1161/STROKEAHA.118.021622

[11] Fletcher, J. L., Makowiecki, K., Cullen, C. L. & Young, K. M. Oligodendrogenesis and myelination regulate cortical development, plasticity and circuit function. *Semin. Cell Dev. Biol.* **118**, 14–23 (2021). 33863642 10.1016/j.semcdb.2021.03.017

[12] Ferrer, I. Oligodendrogliopathy in neurodegenerative diseases with abnormal protein aggregates: the forgotten partner. *Prog. Neurobiol.* **169**, 24–54 (2018). 30077775 10.1016/j.pneurobio.2018.07.004

[13] He, Y. et al. Prosaposin maintains lipid homeostasis in dopamine neurons and counteracts experimental parkinsonism in rodents. *Nat. Commun.* **14**, 5804 (2023). 37726325 10.1038/s41467-023-41539-5 PMC10509278

[14] Meyer, R. C., Giddens, M. M., Schaefer, S. A. & Hall, R. A. GPR37 and GPR37L1 are receptors for the neuroprotective and glioprotective factors prosaptide and prosaposin. *Proc. Natl Acad. Sci. USA* **110**, 9529–9534 (2013). 23690594 10.1073/pnas.1219004110 PMC3677493

[15] Bang, S. et al. Activation of GPR37 in macrophages confers protection against infection-induced sepsis and pain-like behaviour in mice. *Nat. Commun.* **12**, 1704 (2021). 33731716 10.1038/s41467-021-21940-8 PMC7969930

[16] Kamii, H. et al. Amelioration of vasospasm after subarachnoid hemorrhage in transgenic mice overexpressing CuZn-superoxide dismutase. *Stroke* **30**, 867–871 (1999). 10187893 10.1161/01.str.30.4.867

[17] McGinnis, C. S., Murrow, L. M. & Gartner, Z. J. DoubletFinder: doublet detection in single-cell RNA sequencing data using artificial nearest neighbors. *Cell Syst.* **8**, 329–337.e4 (2019). 30954475 10.1016/j.cels.2019.03.003 PMC6853612

[18] Finak, G. et al. MAST: a flexible statistical framework for assessing transcriptional changes and characterizing heterogeneity in single-cell RNA sequencing data. *Genome Biol.* **16**, 278 (2015). 26653891 10.1186/s13059-015-0844-5 PMC4676162

[19] Jin, S. et al. Inference and analysis of cell-cell communication using CellChat. *Nat. Commun.* **12**, 1088 (2021). 33597522 10.1038/s41467-021-21246-9 PMC7889871

[20] Renz, P. et al. Neuroinflammatory reactive astrocyte formation correlates with adverse outcomes in perinatal white matter injury. *Glia* **72**, 1663–1673 (2024). 38924630 10.1002/glia.24575 PMC13502297

[21] Miyamoto, N. et al. The effects of A1/A2 astrocytes on oligodendrocyte linage cells against white matter injury under prolonged cerebral hypoperfusion. *Glia* **68**, 1910–1924 (2020). 32108971 10.1002/glia.23814

[22] Zhou, R. et al. A signalling pathway for transcriptional regulation of sleep amount in mice. *Nature* **612**, 519–527 (2022). 36477534 10.1038/s41586-022-05510-6

[23] Hasegawa, E. et al. Rapid eye movement sleep is initiated by basolateral amygdala dopamine signaling in mice. *Science* **375**, 994–1000 (2022). 35239361 10.1126/science.abl6618

[24] Senzai, Y. & Scanziani, M. A cognitive process occurring during sleep is revealed by rapid eye movements. *Science* **377**, 999–1004 (2022). 36007021 10.1126/science.abp8852 PMC9933572

[25] Markov, N. T. et al. Anatomy of hierarchy: feedforward and feedback pathways in macaque visual cortex. *J. Comp. Neurol.* **522**, 225–259 (2014). 23983048 10.1002/cne.23458 PMC4255240

[26] Mejias, J. F., Murray, J. D., Kennedy, H. & Wang, X. J. Feedforward and feedback frequency-dependent interactions in a large-scale laminar network of the primate cortex. *Sci. Adv.* **2**, e1601335 (2016). 28138530 10.1126/sciadv.1601335 PMC5262462

[27] Sherman, S. M. Thalamus plays a central role in ongoing cortical functioning. *Nat. Neurosci.* **19**, 533–541 (2016). 27021938 10.1038/nn.4269

[28] Melief, E. J. et al. Loss of glutamate signaling from the thalamus to dorsal striatum impairs motor function and slows the execution of learned behaviors. *NPJ Parkinsons Dis.* **4**, 23 (2018). 30083593 10.1038/s41531-018-0060-6 PMC6072777

[29] Oh, S. W. et al. A mesoscale connectome of the mouse brain. *Nature* **508**, 207–214 (2014). 24695228 10.1038/nature13186 PMC5102064

[30] Bonetto, G., Belin, D. & Karadottir, R. T. Myelin: a gatekeeper of activity-dependent circuit plasticity? *Science* **374**, eaba6905 (2021). 34618550 10.1126/science.aba6905

[31] Gibson, E. M. et al. Neuronal activity promotes oligodendrogenesis and adaptive myelination in the mammalian brain. *Science* **344**, 1252304 (2014). 24727982 10.1126/science.1252304 PMC4096908

[32] Steadman, P. E. et al. Disruption of oligodendrogenesis impairs memory consolidation in adult mice. *Neuron* **105**, 150–164.e6 (2020). 31753579 10.1016/j.neuron.2019.10.013 PMC7579726

[33] Simons, M., Gibson, E. M. & Nave, K. A. Oligodendrocytes: myelination, plasticity, and axonal support. *Cold Spring Harb. Perspect. Biol.* **16**, a041359 (2024). 38621824 10.1101/cshperspect.a041359 PMC11444305

[34] Sun, Y. & Grabowski, G. A. Altered autophagy in the mice with a deficiency of saposin A and saposin B. *Autophagy* **9**, 1115–1116 (2013). 23697974 10.4161/auto.24919 PMC3722325

[35] Ma, Q. et al. Oligodendrocytes drive neuroinflammation and neurodegeneration in Parkinson’s disease via the prosaposin-GPR37-IL-6 axis. *Cell Rep.* **44**, 115266 (2025). 39913287 10.1016/j.celrep.2025.115266

[36] Lapitskaya, N. et al. Abnormal corticospinal excitability in patients with disorders of consciousness. *Brain Stimul.* **6**, 590–597 (2013). 23403267 10.1016/j.brs.2013.01.002

[37] Liu, J. et al. Frequency-selective control of cortical and subcortical networks by central thalamus. *eLife* **4**, e09215 (2015). 26652162 10.7554/eLife.09215 PMC4721962

[38] Macdonald, R. L. Subarachnoid Hemorrhage and Loss of Consciousness. *JAMA Neurol.* **73**, 17–18 (2016). 26551437 10.1001/jamaneurol.2015.3485

[39] Provencio, J. J. et al. Neutrophil depletion after subarachnoid hemorrhage improves memory via NMDA receptors. *Brain Behav. Immun.* **54**, 233–242 (2016). 26872422 10.1016/j.bbi.2016.02.007 PMC4828315

[40] Altay, O. et al. Isoflurane attenuates blood-brain barrier disruption in ipsilateral hemisphere after subarachnoid hemorrhage in mice. *Stroke* **43**, 2513–2516(2012). 22773559 10.1161/STROKEAHA.112.661728 PMC3429639

[41] Xu, L. et al. Evaluating brain activity in patients with chronic disorders of consciousness after traumatic brain injury using EEG microstate analysis during hyperbaric oxygen therapy. *CNS Neurosci. Ther.* **31**, e70220 (2025). 39834088 10.1111/cns.70220 PMC11746936

[42] Furutani, N. et al. Utility of complexity analysis in electroencephalography and electromyography for automated classification of sleep-wake states in mice. *Sci. Rep.* **15**, 3080 (2025). 39856071 10.1038/s41598-024-74008-0 PMC11760340

[43] Yamada, R. G., Matsuzawa, K., Ode, K. L. & Ueda, H. R. An automated sleep staging tool based on simple statistical features of mice electroencephalography (EEG) and electromyography (EMG) data. *Eur. J. Neurosci.* **60**, 5467–5486 (2024). 39072800 10.1111/ejn.16465

[44] Forgacs, P. B. et al. Corticothalamic connectivity in aneurysmal subarachnoid hemorrhage: relationship with disordered consciousness and clinical outcomes. *Neurocrit. Care* **36**, 760–771 (2022). 34669180 10.1007/s12028-021-01354-6

[45] Sheng, B. et al. Exosomes-mediated delivery of miR-486-3p alleviates neuroinflammation via SIRT2-mediated inhibition of mitophagy after subarachnoid hemorrhage. *Stroke Vasc. Neurol.* **10**, 335–346 (2025). 39357894 10.1136/svn-2024-003509 PMC12285680

[46] Peng, H. et al. Risk factors for in-hospital seizures of aneurysmal subarachnoid hemorrhage after endovascular treatment: a real-world study. *World Neurosurg.* **188**, e480–e490 (2024). 38815925 10.1016/j.wneu.2024.05.140

[47] Giacino, J., Fins, J. J., Machado, A. & Schiff, N. D. Central thalamic deep brain stimulation to promote recovery from chronic posttraumatic minimally conscious state: challenges and opportunities. *Neuromodulation* **15**, 339–349 (2012). 22624587 10.1111/j.1525-1403.2012.00458.x

[48] Schiff, N. D. Central thalamic deep brain stimulation to support anterior forebrain mesocircuit function in the severely injured brain. *J. Neural. Transm.* **123**, 797–806 (2016). 27113938 10.1007/s00702-016-1547-0

[49] Salvalaggio, A. et al. Post-stroke deficit prediction from lesion and indirect structural and functional disconnection. *Brain* **143**, 2173–2188 (2020). 32572442 10.1093/brain/awaa156 PMC7363494

[50] Wu, Y., Peng, J., Pang, J., Sun, X. & Jiang, Y. Potential mechanisms of white matter injury in the acute phase of experimental subarachnoid haemorrhage. *Brain* **140**, e36 (2017). 28430870 10.1093/brain/awx084

[51] Peng, J. et al. Toll-like receptor 4-mediated microglial inflammation exacerbates early white matter injury following experimental subarachnoid hemorrhage. *J. Neurochem.* **166**, 280–293 (2023). 37309616 10.1111/jnc.15851

[52] O’Brien, J. S. et al. Identification of the neurotrophic factor sequence of prosaposin. *FASEB J.* **9**, 681–685 (1995). 7768361 10.1096/fasebj.9.8.7768361

[53] Vaccaro, A. M., Salvioli, R., Tatti, M. & Ciaffoni, F. Saposins and their interaction with lipids. *Neurochem. Res.* **24**, 307–314 (1999). 9972880 10.1023/a:1022530508763

[54] Hiraiwa, M., Campana, W. M., Mizisin, A. P., Mohiuddin, L. & O’Brien, J. S. Prosaposin: a myelinotrophic protein that promotes expression of myelin constituents and is secreted after nerve injury. *Glia* **26**, 353–360 (1999). 10383054

[55] Meyer, R. C., Giddens, M. M., Coleman, B. M. & Hall, R. A. The protective role of prosaposin and its receptors in the nervous system. *Brain Res.* **1585**, 1–12 (2014). 25130661 10.1016/j.brainres.2014.08.022 PMC4529117

[56] Wu, Z. et al. Single-cell analysis of spinal cord injury reveals functional heterogeneity of oligodendrocyte lineage cells. *Gene* **886**, 147713 (2023). 37579960 10.1016/j.gene.2023.147713

[57] Qian, Z. et al. Osteocalcin attenuates oligodendrocyte differentiation and myelination via GPR37 signaling in the mouse brain. *Sci. Adv.* **7**, eabi5811 (2021). 34678058 10.1126/sciadv.abi5811 PMC8535816

[58] Bugiani, M., Plug, B. C., Man, J. H. K., Breur, M. & van der Knaap, M. S. Heterogeneity of white matter astrocytes in the human brain. *Acta Neuropathol.* **143**, 159–177 (2022). 34878591 10.1007/s00401-021-02391-3

[59] Zhou, X. et al. Prosaposin facilitates sortilin-independent lysosomal trafficking of progranulin. *J. Cell Biol.* **210**, 991–1002 (2015). 26370502 10.1083/jcb.201502029 PMC4576858

[60] Zhou, X. et al. Impaired prosaposin lysosomal trafficking in frontotemporal lobar degeneration due to progranulin mutations. *Nat. Commun.* **8**, 15277 (2017). 28541286 10.1038/ncomms15277 PMC5477518

[61] Nicholson, A. et al. Prosaposin is a regulator of progranulin levels and oligomerization. *Nat. Commun.* **7**, 11992 (2016). 27356620 10.1038/ncomms11992 PMC4931318

