# Supplementary material for: Oligodendrocyte prosaposin restores subarachnoid haemorrhage-induced consciousness impairment
Source: Exp Mol Med. 2026 Jul 2;58(7):2184–201. doi: 10.1038/s12276-026-01764-6 (PMC13434050; doi:10.1038/s12276-026-01764-6)
Supplement: Supplementary file 1 [file 12276_2026_1764_MOESM1_ESM.pdf]

Supplementary Materials for

Oligodendrocyte Prosaposin Restores Subarachnoid Hemorrhage-Induced Consciousness Impairment

Supplementary information accompanies the manuscript on the Experimental & Molecular Medicine website

The file includes:

Supplementary Fig. 1 – Fig. 10

Supplementary Table 1 and Table 2

Other Supplementary Materials for this manuscript include the following:

Data File S1

Supplementary Fig. 1: Dimensionality reduction and clustering annotation of snRNA-seq data from SAH and sham samples.
Supplementary Fig. 2: Neuronal heterogeneity following SAH in mice.
Supplementary Fig. 3: Lef1 is enriched in thalamic excitatory neurons and acts as a key transcriptional regulator following SAH.
Supplementary Fig. 4: Representative images of neuron cell encapsulation in the CL and mPFC nuclei.
Supplementary Fig. 5: Monocal2 pseudotime analysis of oligodendrocytes.
Supplementary Fig. 6: CellChat analysis of cell-cell communication and cell-type-specific gene expression following SAH.
Supplementary Fig. 7: Expression changes of PSAP in the corpus callosum after SAH and validation of Psap overexpression effects in oligodendrocytes
Supplementary Fig. 8: HuPSAP restores CL→mPFC functional connectivity and ameliorates myelin damage in SAH mice.
Supplementary Fig. 9: Oligodendrocyte-specific Gpr37 knockdown attenuates the therapeutic effects of HuPSAP on consciousness following SAH.
Supplementary Fig. 10: Oligodendrocyte Psap knockdown induces consciousness impairment in Sham mice.

Supplementary Table 1. Patient characteristics.
Supplementary Table 2. Antibodies, Reagents, and Software Resources.

Supplementary Figures

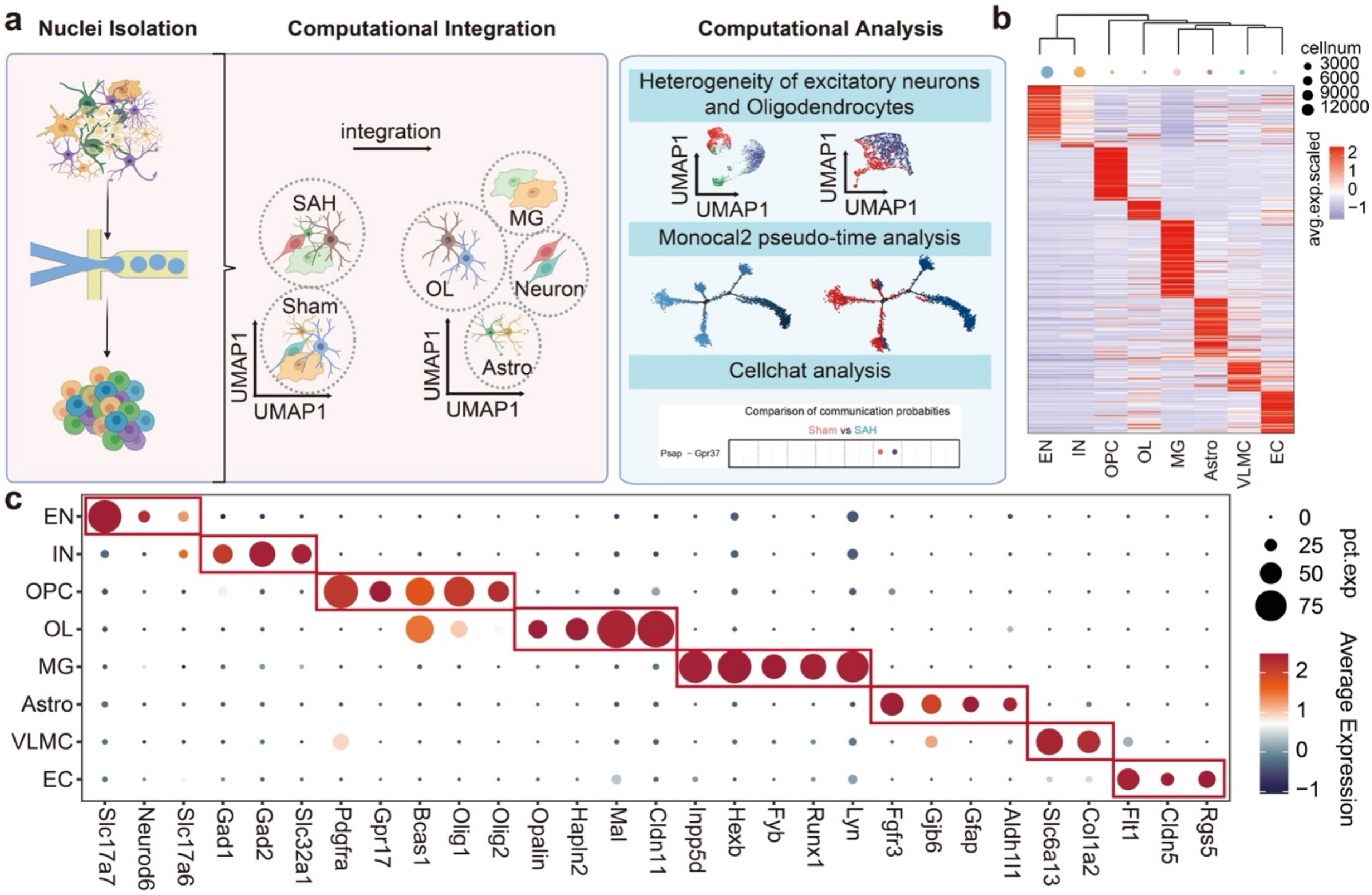

Supplementary Fig. 1. Dimensionality reduction and clustering annotation of snRNA-seq data from SAH and sham samples. (a) Workflow for snRNA-seq sample preparation and data analysis. (b) Heatmap of differentially expressed genes across annotated cell types, with circle size above indicating the number of cells and heatmap color representing the average gene expression level. (c) Bubble plot showing cell type classification based on canonical cell-specific markers, with bubble size indicating the proportion of cells expressing the marker and color representing the average expression level.

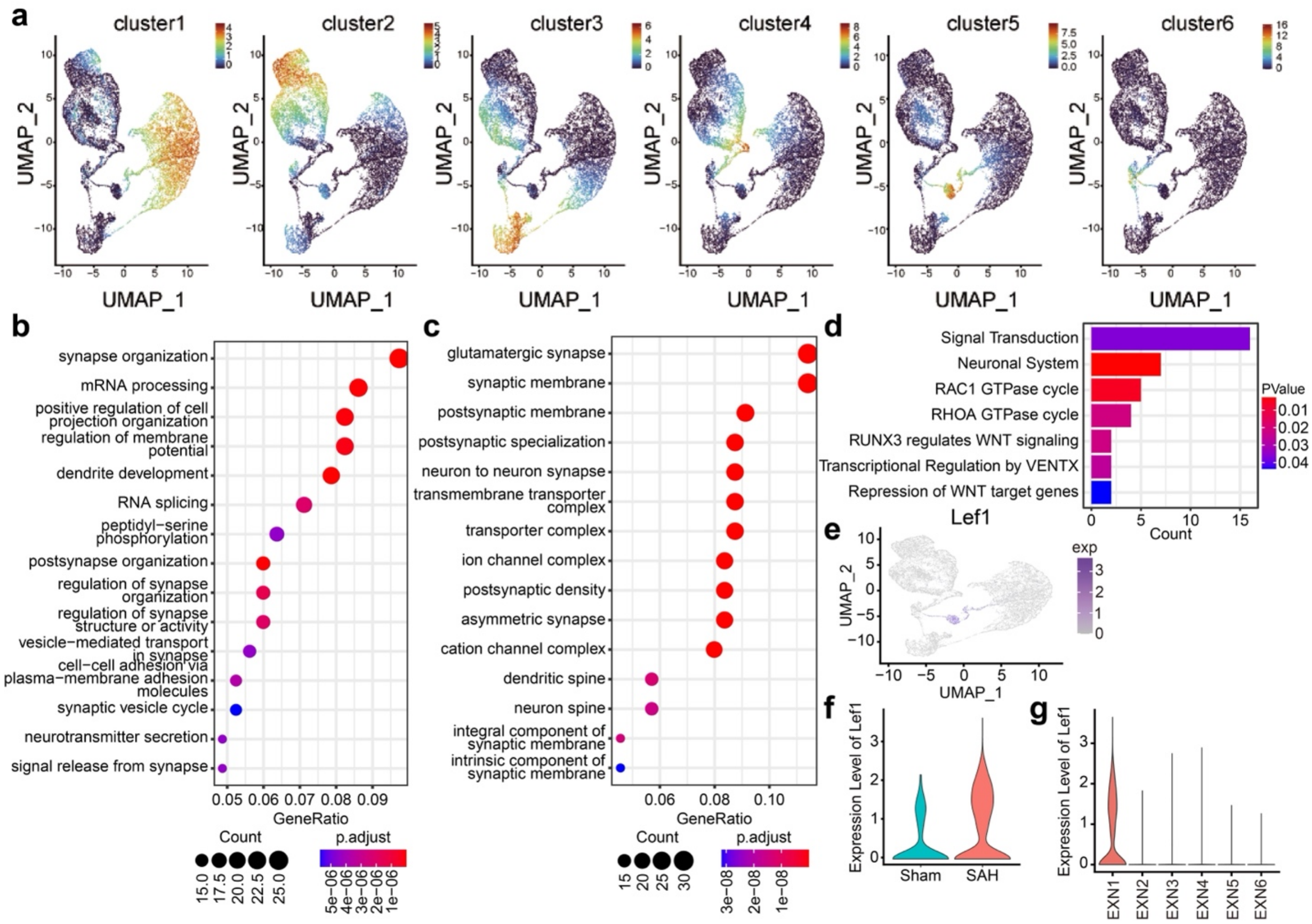

Supplementary Fig. 2. Neuronal heterogeneity following SAH in mice. (a) UMAP plot showing six subclusters of excitatory neurons. (b, c) GO functional enrichment analysis of EXN1 in (b) biological processes and (c) cellular components. (d) Reactome pathway enrichment analysis. (e) UMAP visualization of Lef1 expression in excitatory neurons from snRNA-seq data. (f) Violin plot comparing Lef1 expression levels in EXN1 between the sham and SAH groups. (g) Violin plot showing Lef1 expression levels across excitatory neuron subtypes EXN1-6.

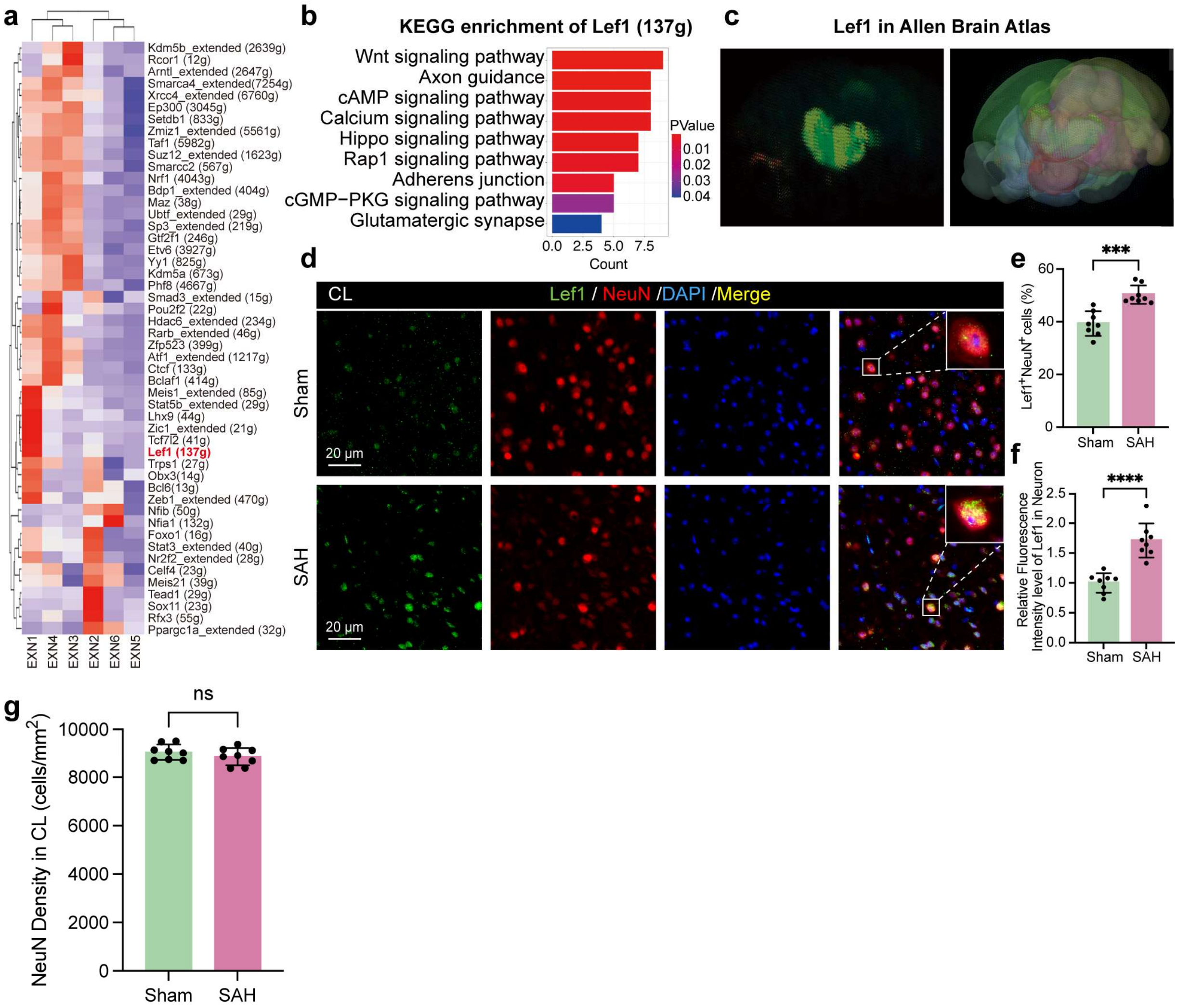

Supplementary Fig. 3. Lef1 is enriched in thalamic excitatory neurons and acts as a key transcriptional regulator following SAH. (a) Heatmap of transcriptional activity for each transcriptional regulatory unit in the EXN1-6 subgroups from SCENIC analysis. (b) KEGG pathway enrichment analysis of Lef1 transcriptional regulatory units. (c) 3D mapping of Lef1 in the Allen Brain Atlas. (d) Representative immunofluorescence images of Lef1 (green), NeuN (red), DAPI (blue), and merged channels in the central lateral thalamus (CL) of sham and SAH mice. Right panels show high-magnification images of the boxed regions. Scale bars: 20 µm. (e) Quantification of the percentage of Lef1+NeuN+ cells among total NeuN+ cells in the CL of sham and SAH mice (n = 8, ****p* < 0.001, unpaired t-test). (f) Quantification of relative fluorescence intensity of Lef1 in NeuN-positive neurons in the CL of sham and SAH mice (n = 8, *****p* < 0.0001, unpaired t-test). (g) Quantification of NeuN-positive neuronal density (cells/mm²) in the CL of sham and SAH mice (n = 8, ns = not significant, unpaired t-test).

a

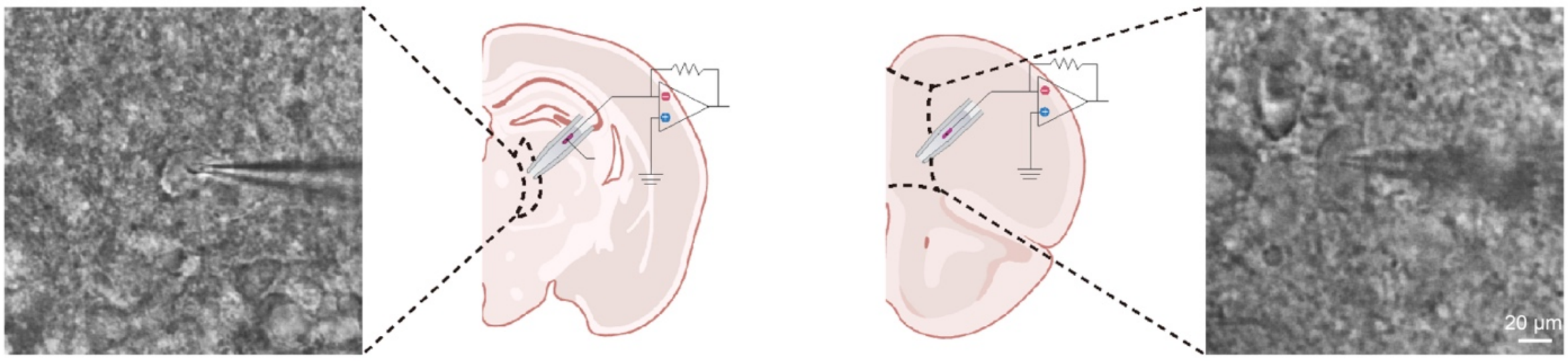

Supplementary Fig. 4. Representative images of neuron cell encapsulation in the CL and mPFC nuclei; scale bar =20 μm.

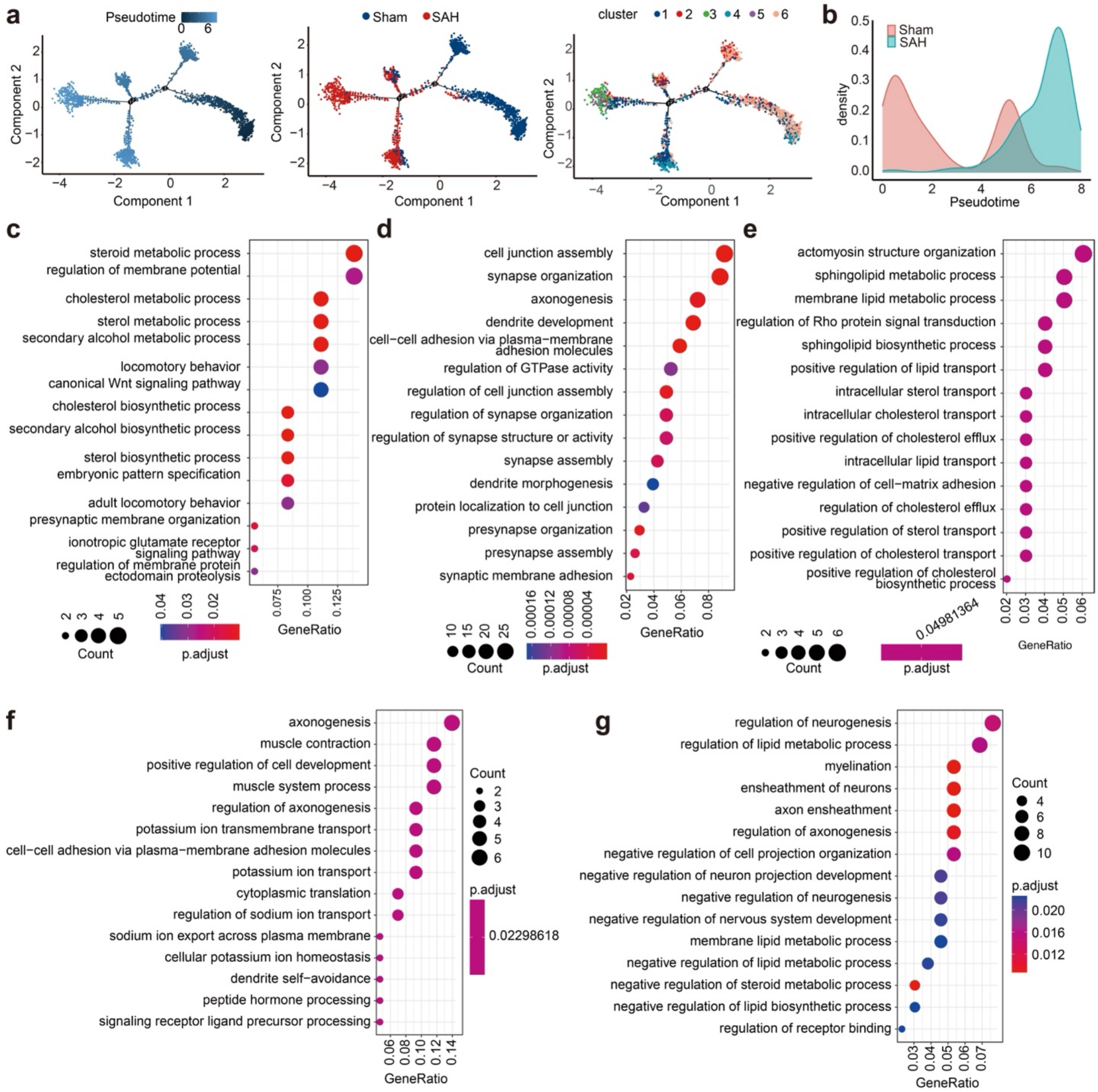

Supplementary Fig. 5. Monocal2 pseudotime analysis of oligodendrocytes. (a) Monocle2 trajectory analysis of oligodendrocytes shows clear separation between the sham and SAH groups after State 1. (b) Pseudotime analysis of oligodendrocyte differentiation trajectories, highlighting developmental transitions post-SAH. (c-g) GO functional enrichment analysis of differentially expressed genes in the 5 subgroups from pseudotime analysis.

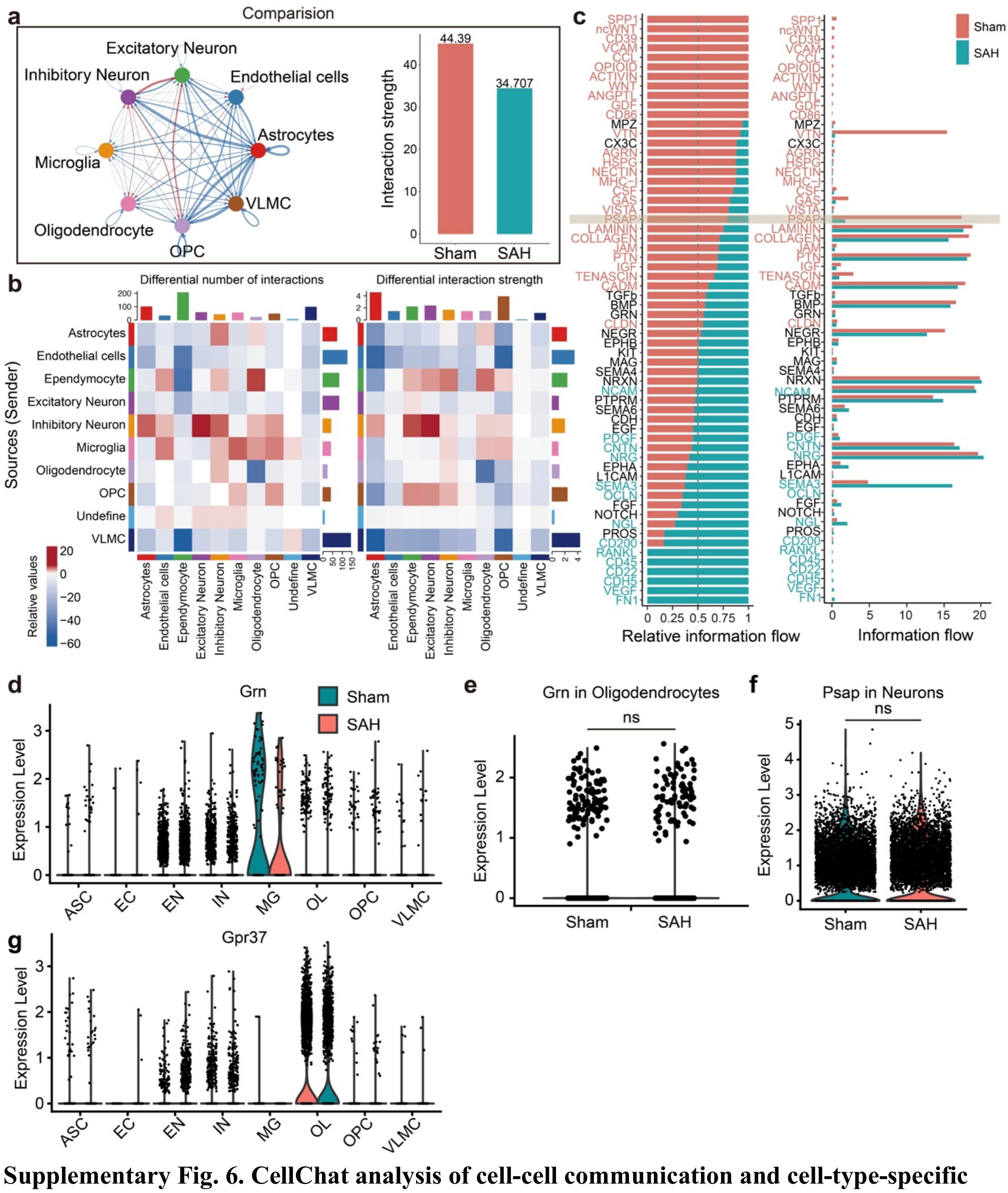

Supplementary Fig. 6. CellChat analysis of cell-cell communication and cell-type-specific gene expression following SAH. (a) Left: Chord diagram comparing cell interaction strength between Sham and SAH groups, with blue representing a decrease in interaction strength after SAH and red representing an increase. Right: Bar chart comparing interaction strength. (b) Heatmap comparing interaction strength between different cell types in Sham and SAH, with red indicating an increase after SAH and blue indicating a decrease. (c) Proportion of all interaction pathways in the Sham and SAH groups. (d) Violin plots showing Grn (Granulin) expression

levels across different cell types in Sham and SAH groups. (e) Quantification of Grn expression levels specifically in oligodendrocytes comparing Sham and SAH groups. (f) Quantification of Psap expression levels specifically in neurons comparing Sham and SAH groups. (g) Violin plots showing Gpr37 expression levels across different cell types.

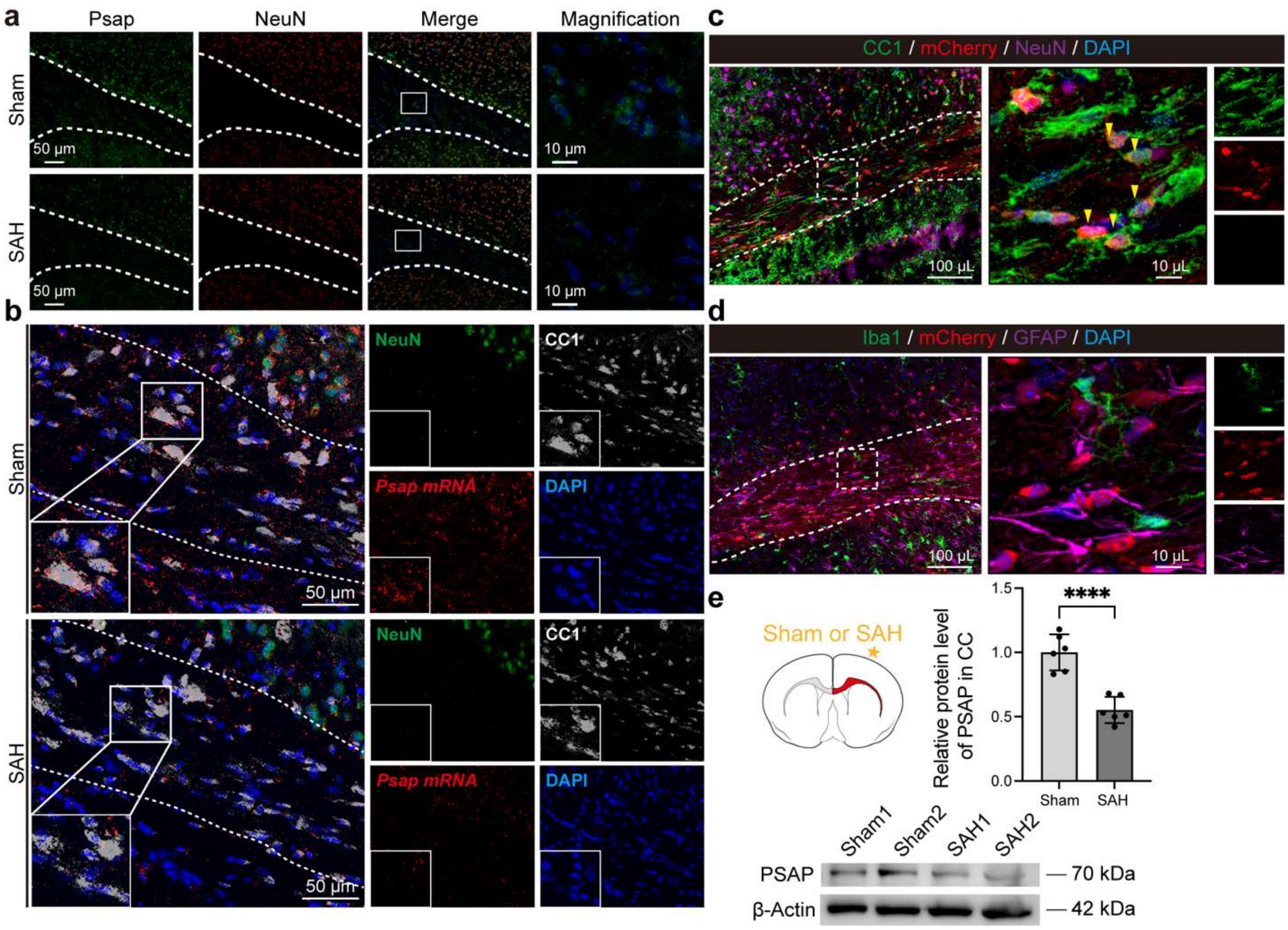

Supplementary Fig. 7. Expression changes of PSAP in the corpus callosum after SAH and validation of Psap overexpression effects in oligodendrocytes. (a) Representative immunofluorescence images of Psap (green) and NeuN (red) co-staining in the corpus callosum (CC) of sham and SAH mice. Dashed lines outline the CC boundaries. Right panels show high-magnification images of the boxed regions. Scale bars: 50 µm (left panels) and 10 µm (right panels). (b) Representative images of RNAscope fluorescent in situ hybridization (FISH) for *Psap* mRNA (red) combined with immunofluorescence staining for the mature oligodendrocyte marker CC1 (white), the neuronal marker NeuN (green), and DAPI (blue) in the corpus callosum of sham and SAH mice. Dashed lines outline the corpus callosum boundaries. Right panels show individual single-channel images of the full field. Insets show high-magnification views of representative CC1-positive oligodendrocytes. Scale bars: 50 µm. (c) Representative immunofluorescence images showing co-localization of Psap-overexpressing virus (mCherry, red) with CC1 (oligodendrocyte marker, green) and NeuN (neuronal marker, magenta) in the corpus callosum. DAPI (blue) marks nuclei. Dashed lines outline the CC. Yellow arrowheads indicate mCherry-positive cells co-labeled with CC1, confirming oligodendrocyte-specific viral transduction. Scale bars: 100 µm (left) and 10 µm (right). (d) Representative immunofluorescence images of Iba1 (green), mCherry (red), GFAP (magenta), and DAPI (blue) in the corpus callosum. Scale bars: 100 µm (left) and 10 µm (right). (e) Western blot analysis of PSAP protein levels in the corpus callosum of sham and SAH mice. Right: Quantification of relative PSAP protein levels normalized to β-actin (n = 6, ****$p < 0.0001$, unpaired t-test).

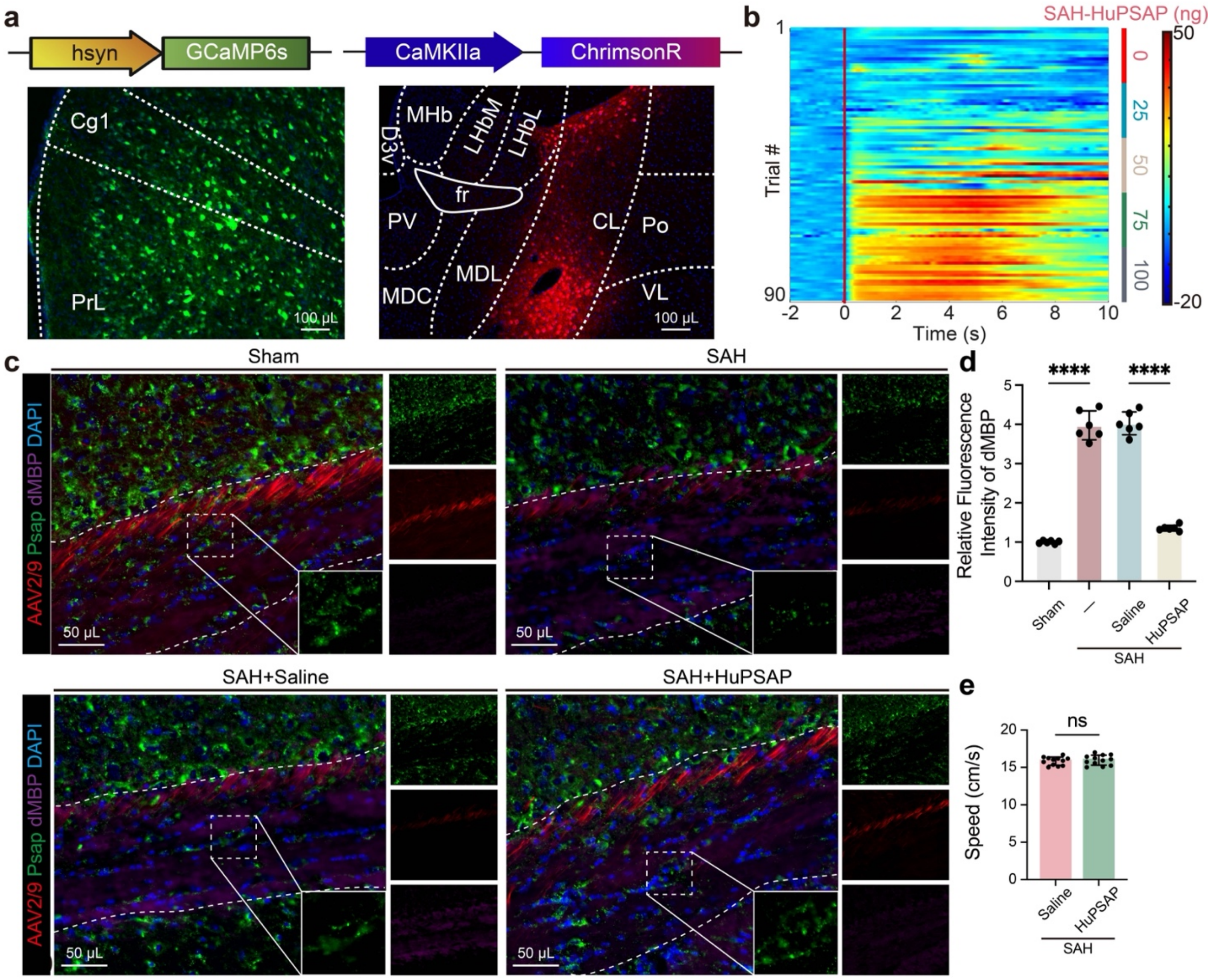

Supplementary Fig. 8. HuPSAP restores CL→mPFC functional connectivity and ameliorates myelin damage in SAH mice. (a) Representative images showing the transfection of the light-sensitive protein ChrimsonR and the calcium indicator GCaMP6s, with boundaries outlined by white dashed lines. Scale bar: 100 µm. (b) Heatmap of ΔF/F calcium signals across different HuPSAP concentration groups. (c) AAV2/9-hSyn-mCherry-labeled CL axons (red) in the CC co-stained for Psap (green) and dMBP (magenta). Scale bars: 50 µm. (d) Quantification of relative fluorescence intensities of dMBP levels in mCherry-positive regions of the CC (n = 6, ****$p < 0.0001$, one-way ANOVA). (e) Quantitative analysis of swimming speed during the Morris water maze and probe tests (n = 12, ns = not significant, Unpaired t test).

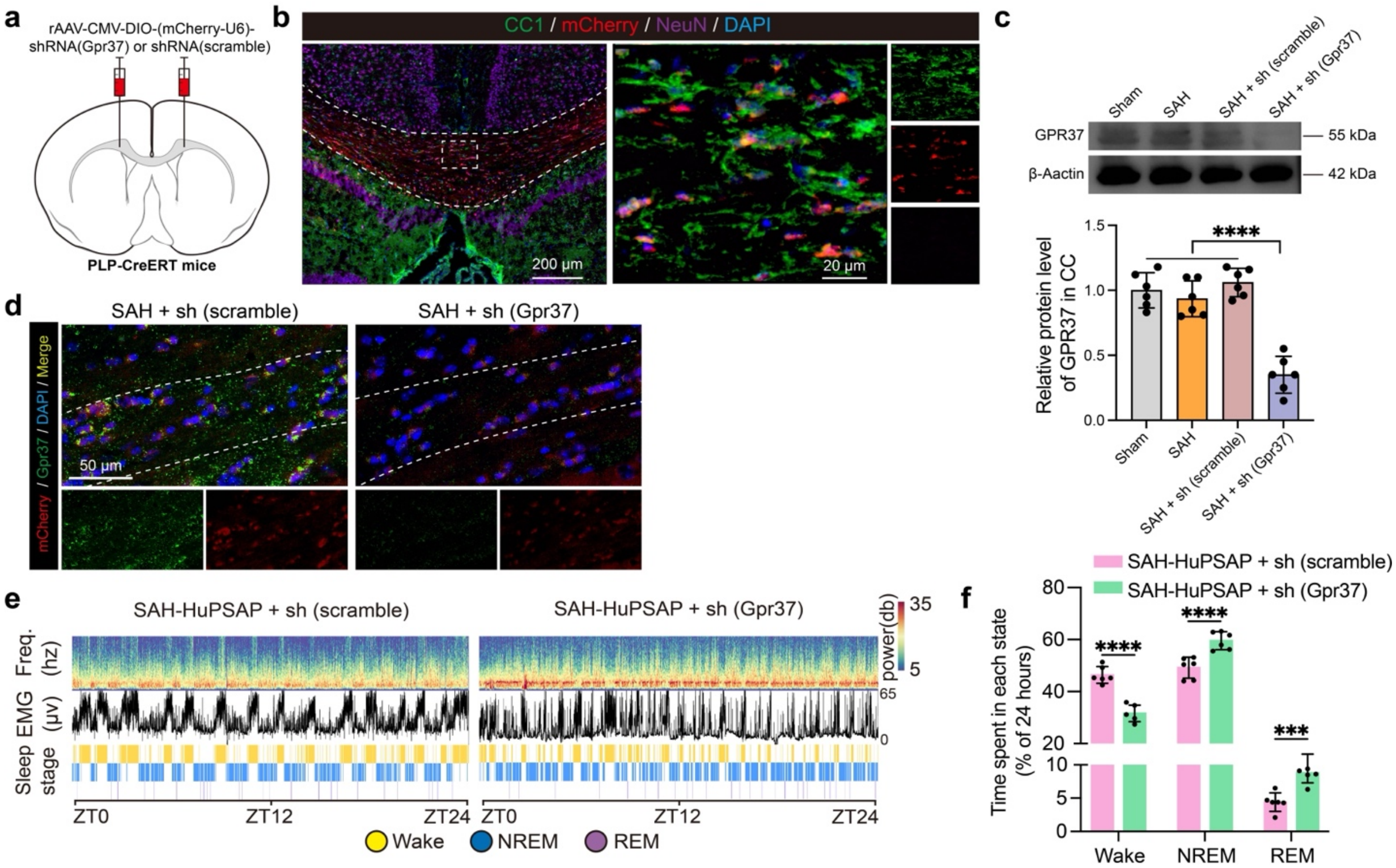

Supplementary Fig. 9. Oligodendrocyte-specific Gpr37 knockdown attenuates the therapeutic effects of HuPSAP on consciousness following SAH. (a) Schematic diagram of the experimental strategy for oligodendrocyte-specific Gpr37 knockdown. Cre-dependent AAV vectors [rAAV-CMV-DIO-(mCherry-U6)-shRNA] carrying either Gpr37 shRNA or scramble control shRNA were bilaterally injected into the corpus callosum of PLP-CreERT mice, enabling selective knockdown of Gpr37 in oligodendrocytes upon tamoxifen induction. (b) Representative immunofluorescence images validating oligodendrocyte-specific viral transduction. Scale bars: 200 μm (left) and 20 μm (middle). (c) Western blot validation of Gpr37 knockdown efficiency. Bottom: Quantification of relative GPR37 protein levels normalized to β-actin. (d) Representative immunofluorescence images validating Gpr37 knockdown at the cellular level. Scale bar: 50 μm. (e) Representative EEG spectrograms (top), EMG traces (middle), and hypnograms showing sleep-wake staging (bottom) over a 24-hour recording period in SAH mice treated with HuPSAP combined with either scramble shRNA [SAH-HuPSAP+sh(scramble)] or Gpr37 shRNA [SAH-HuPSAP+sh(Gpr37)]. (f) Quantification of time spent in each vigilance state (Wake, NREM, and REM) as a percentage of 24 hours (n = 6, ***$p < 0.001$, ****$p < 0.0001$). Data are presented as mean ± SD.

.

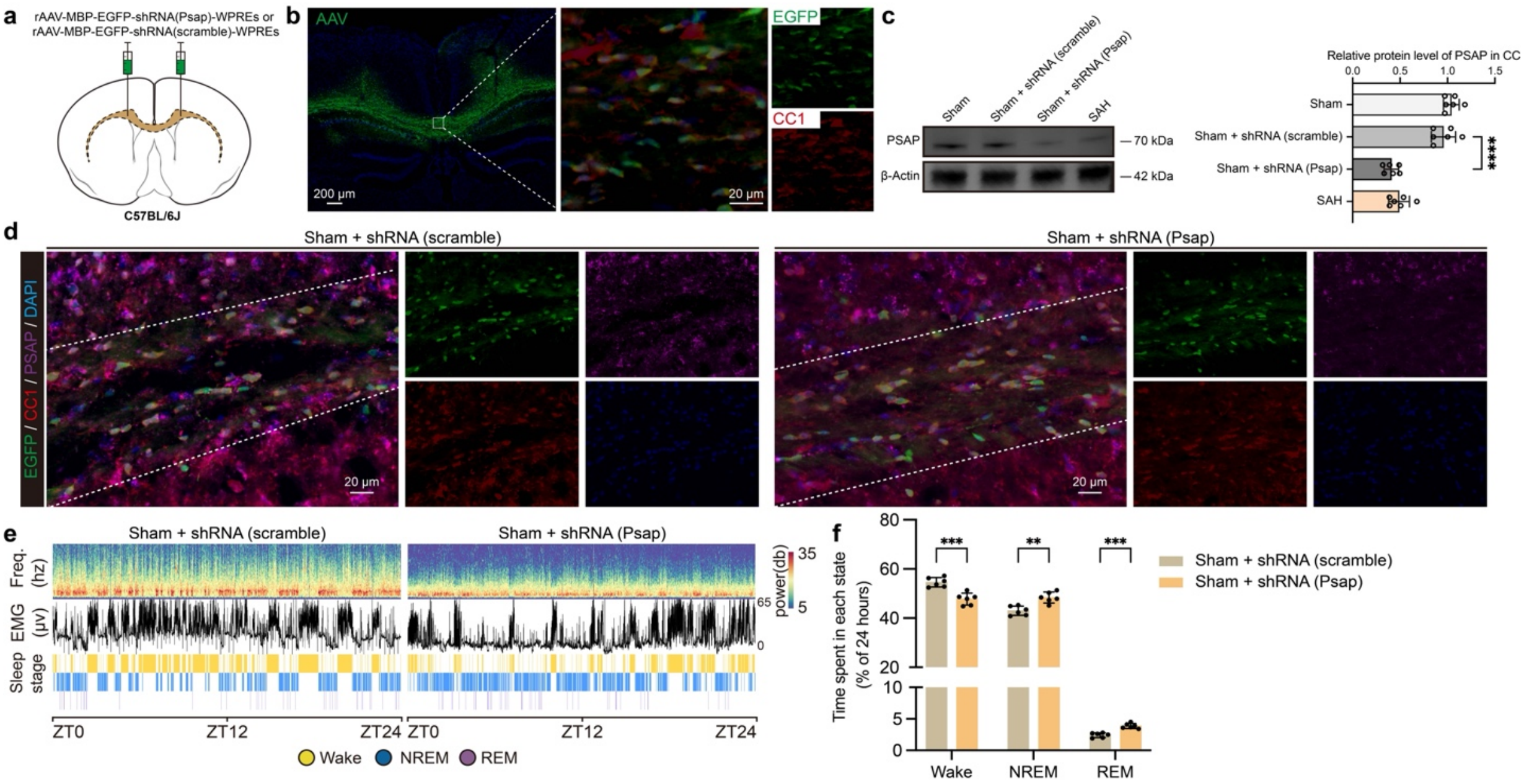

Supplementary Fig. 10. Oligodendrocyte Psap knockdown induces consciousness impairment in sham mice. (a) Schematic of bilateral stereotaxic injection of MBP promoter-driven AAV vectors carrying either Psap shRNA (rAAV-MBP-EGFP-shRNA (Psap)-WPREs) or scramble control shRNA (rAAV-MBP-EGFP-shRNA(scramble)-WPREs) into the corpus callosum of C57BL/6J mice. (b) Left: Representative low-magnification fluorescence image showing AAV transfection (green) in the corpus callosum. Dashed lines outline the corpus callosum. Right: High-magnification images of the boxed region showing co-localization of EGFP (green) with the mature oligodendrocyte marker CC1 (red), confirming oligodendrocyte-specific viral transduction. Scale bars: 200 μm (left) and 20 μm (right). (c) Left: Western blot analysis of PSAP protein levels in the corpus callosum across four groups: Sham, Sham + shRNA(scramble), Sham + shRNA (Psap), and SAH. β-Actin served as the loading control. Right: Quantification of relative PSAP protein levels normalized to β-Actin (n = 6, ****$p <$ 0.0001). (d) Representative immunofluorescence images of EGFP (green), CC1 (red), PSAP (magenta), and DAPI (blue) in the corpus callosum of Sham + shRNA(scramble) and Sham + shRNA (Psap) groups. Dashed lines outline the corpus callosum. Right panels show individual single-channel images of the boxed regions. PSAP immunofluorescence is visibly reduced in CC1-positive oligodendrocytes in the Psap knockdown group. Scale bars: 20 μm. (e) Representative EEG spectrograms (top), EMG traces (middle), and hypnograms showing sleep-wake staging (bottom) over a 24-hour recording period in Sham + shRNA(scramble) and Sham + shRNA (Psap) mice. (f) Quantification of time spent in each vigilance state (Wake, NREM, and REM) as a percentage of 24 hours (n = 6, **$p < 0.01$). Data are presented as mean ± SD.

Supplementary Table 1. Patient characteristics.

Patient no.	Age	Gender	cause of disease	Amount of bleeding	Hunt and Hess scale	Sample type
1	66	M	anterior communicating artery aneurysm	10 mL	2	brain tissue
2	72	F	anterior communicating artery aneurysm	30 mL	4	brain tissue
3	26	M	anterior communicating artery aneurysm	15 mL	2	brain tissue
4	71	F	anterior communicating artery aneurysm	40 mL	4	brain tissue
5	51	F	multiple aneurysms	10 mL	2	brain tissue
6	68	M	anterior communicating artery aneurysm	10 mL	2	brain tissue
7	42	F	multiple aneurysms aneurysm	20 mL	3	plasma
8	41	M	middle cerebral artery aneurysm	15 mL	2	plasma
9	60	M	anterior communicating artery aneurysm	10 mL	2	plasma
10	40	M	middle cerebral artery aneurysm	20 mL	3	plasma
11	39	F	anterior communicating artery aneurysm	10ml	2	plasma
12	51	M	anterior communicating artery	10 mL	2	plasma

			aneurysm			
13	65	F	temporal lobe tumor	-	-	brain tissue and plasma
14	446	F	temporoparietal glioma	-	-	brain tissue and plasma
15	73	M	frontal lobe malignancy	-	-	brain tissue and plasma
16	69	F	Frontal lobe glioma	-	-	brain tissue and plasma
17	57	F	brain metastasis from lung cancer	-	-	brain tissue and plasma
18	60	M	Insular glioma	-	-	brain tissue and plasma

Note: M, male; F, female;

Supplementary Table 2. Antibodies, Reagents, and Software Resources.

REAGENT OR RESOURCE	SOURCE	IDENTIFIER
Antibodies		
Mouse anti-MBP	Abcam	Cat# ab11159
Rabbit anti-dMBP	Merckmillipore	Cat# AB5864
Rabbit anti-Psap	Abcam	Cat# ab300469
Rabbit anti-Gpr37	Proteintech Group	Cat# 14820-1-AP
Mouse anti-Psap	Proteintech Group	Cat# 66473-1-Ig
Mouse anti-CC1	Abcam	Cat# ab16794
Mouse anti- Gpr37	Santa Cruz	Cat# sc-390110
Rabbit anti-Lef1	Abcam	Cat# ab137872
Mouse anti-NeuN	Abcam	Cat# ab104224
Donkey anti-rabbit IgG （H+L） Secondary Antibody, Alexa Fluor™ 488	Thermo Fisher Scientific	Cat# A-21206
Donkey anti-mouse IgG （H+L） Secondary Antibody, Alexa Fluor™ 555	Thermo Fisher Scientific	Cat# A-31570
Donkey anti-Rabbit IgG (H+L) Highly Cross-Adsorbed Secondary Antibody, Alexa Fluor™ 555	Thermo Fisher Scientific	Cat# A-31572
Donkey anti-Mouse IgG (H+L) Highly Cross-Adsorbed Secondary Antibody, Alexa Fluor™ 488	Thermo Fisher Scientific	Cat# A-21202
Donkey anti-Rabbit IgG (H+L) Highly Cross-Adsorbed Secondary Antibody, Alexa Fluor™ 647	Thermo Fisher Scientific	Cat# A-31573
Bacterial and virus strains		
HSV-tdTomato	BrainVTA	H01002
AAV-hSyn-EGFP(2/Re)	BrainVTA	PT-1990
AAV-CaMKIIα-ChrimsonR	BrainVTA	PT-4285
AAV-hSyn-GCaMP6s	BrainVTA	PT-0145
AAV-MBP-Psap-mCherry	BrainVTA	PT-1515

rAAV-MBP-EGFP-shRNA(Psap)-WPREs	BrainVTA	PT-16167
AAV2/9-hsyn-mCherry	BrainVTA	PT-1008
Chemicals, peptides, and recombinant proteins		
Recombinant human PSAP protein	Abcam	ab167924
Experimental models: Organisms/strains		
C57BL/6J mice	Ziyuan	NA
Critical commercial assays		
Mouse PSAP (Prosaposin) ELISA Kit	Finetest	EM2515
Human PSAP(Prosaposin) ELISA Kit	Finetest	EH3669
Duolink® flowPLA Mouse/Rabbit Starter Kit - Green	MilliporeSigma	DUO94102
Software and algorithms		
MATLAB R2015b	MathWorks	https://www.mathworks.com/products/matlab.html
Prism	GraphPad Software	https://www.graphpad.com/scientific-software/prism
ImageJ	National Institutes of Health	https://imagej.nih.gov/ij/index.html
Lunion stage	LunionData	https://www.luniondata.com/lunion-stage
Analysis code	Li et al.	https://doi.org/10.5281/zenodo.15275572
Other		
Mouse EEG & EMG Recording System	QUANLAN	https://www.eegion.com
Optogenetic recording system	ThinkerTech	http://www.thinkerbiotech.com/
